# 1,3-Butadiynamides the Ethynylogous Ynamides: Synthesis, Properties and Applications in Heterocyclic Chemistry

**DOI:** 10.3390/molecules28114564

**Published:** 2023-06-05

**Authors:** Illia Lenko, Carole Alayrac, Igor Bożek, Bernhard Witulski

**Affiliations:** Laboratoire de Chimie Moléculaire et Thio-Organique (LCMT), CNRS UMR 6507, ENSICAEN & UNICAEN, Normandie University, 6 Bd Maréchal Juin, 14050 Caen, France

**Keywords:** 1,3-diynamides, ynamides, alkynes, homogeneous catalysis, heterocycles, annulation reactions, cycloaddition cascade reactions, helical twisted frontier molecular orbitals (Hel-FMO), Hexa-dehydro-Diels–Alder (HDDA) reaction, quinolines, indoles, carbazoles

## Abstract

1,3-butadiynamides—the ethynylogous variants of ynamides—receive considerable attention as precursors of complex molecular scaffolds for organic and heterocyclic chemistry. The synthetic potential of these C4-building blocks reveals itself in sophisticated transition-metal catalyzed annulation reactions and in metal-free or silver-mediated HDDA (Hexa-dehydro-Diels–Alder) cycloadditions. 1,3-Butadiynamides also gain significance as optoelectronic materials and in less explored views on their unique helical twisted frontier molecular orbitals (Hel-FMOs). The present account summarizes different methodologies for the synthesis of 1,3-butadiynamides followed by the description of their molecular structure and electronic properties. Finally, the surprisingly rich chemistry of 1,3-butadiynamides as versatile C4-building blocks in heterocyclic chemistry is reviewed by compiling their exciting reactivity, specificity and opportunities for organic synthesis. Besides chemical transformations and use in synthesis, a focus is set on the mechanistic understanding of the chemistry of 1,3-butadiynamides—suggesting that 1,3-butadiynamides are not just simple alkynes. These ethynylogous variants of ynamides have their own molecular character and chemical reactivity and reflect a new class of remarkably useful compounds.

## 1. Introduction

**Figure 1:**
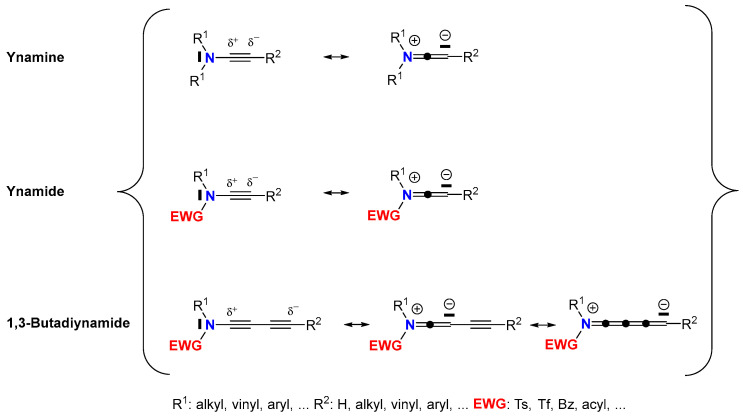
Mesomeric resonance structures of ynamines, ynamides and 1,3-butadiynamides.

Since the disclosure of a first reliable and widely applicable synthesis of ynamides (*N*-alkynylamides) in 1998 [1], these functionalized acetylenes combining the diverse reactivity of a carbon–carbon triple bond with one of the most important functional groups of organic chemistry—the amino function—found widespread interest within the chemical community. Ynamides distinguish from their long-time known electron-rich variants—the ynamines (*N*-alkynylamines)—by having at least one electron-withdrawing group (EWG) attached to the nitrogen heteroatom. This EWG is capable of serving as a protective group in further chemical manipulations—but most importantly—it guarantees a decent chemical stability of the otherwise electron-rich acetylenic unit by lowering its HOMO. As such, the nitrogen bound EWG decreases the electron density of the adjacent alkyne moiety by inducing a more or less—but significantly—polarized carbon–carbon triple bond (Figure 1). Consequently, this results in tuning reactivity and selectivity. Ynamides are habitually bench-stable functionalized acetylenes that withstand an aqueous work-up procedure as well as purification by column chromatography on silica gel—both important features that are required for being useful molecular building blocks in organic synthesis. Over the years, the chemistry of ynamides became well-recognized. Ynamides nowadays serve as multipurpose substrates in organic synthesis including transition metal, ionic, radical and photoredox-mediated processes leading to *N*-heterocyclic compounds, natural products and other molecular targets for life and material sciences. Their exceptional rich and diverse chemistry covers several review articles [2,3,4,5,6,7,8,9,10,11,12,13,14,15,16].

More recently, the ethynylogous variants of ynamides—the 1,3-butadiynamides—likewise received considerable attention in organic and heterocyclic chemistry. They found interesting use in transition metal-catalyzed annulation reactions, like the Hexa-dehydro-Diels–Alder (HDDA) reaction [17,18,19,20,21], and as optoelectronic materials due to their underlying push–pull character. Notably, the intrinsic polarization of ynamides is not only preserved in 1,3-butadiynamides. It is furthermore extendable via the ethynylogous principle from a 1,2- to a formal 1,4-polarization. Therefore, 1,3-butadiynamides are appealing for nitrogen-functionalized C4-building blocks for the construction of complex structural motifs. As both triple bonds can be simultaneously or consecutively functionalized via an electrophilic and/or nucleophilic reaction step, the selective introduction of more than one substituent might be achievable selectively. Other important features of molecular 1,3-butadiynamide scaffolds are their facile synthesis and their often superb stability. This not only accounts for 1,4-diynamides, but also for the higher conjugated oligoynamides. Some chemistry associated with 1,3-butadiynamides has been briefly mentioned in reviews covering synthesis and use of ynamines and ynamides [3,4,5,12,13,14,15,16]. However, there is so far no comprehensive article focusing on the ethynylogous variant of ynamides—the 1,3-butadiynamides. Highlighting the chemistry of 1,3-butadiynamides and compiling their reactivity, specificity and miscellaneous synthetic potential is the purpose of our present review.

## 2. Synthesis of 1,3-Butadiynamides

**Scheme 1:**
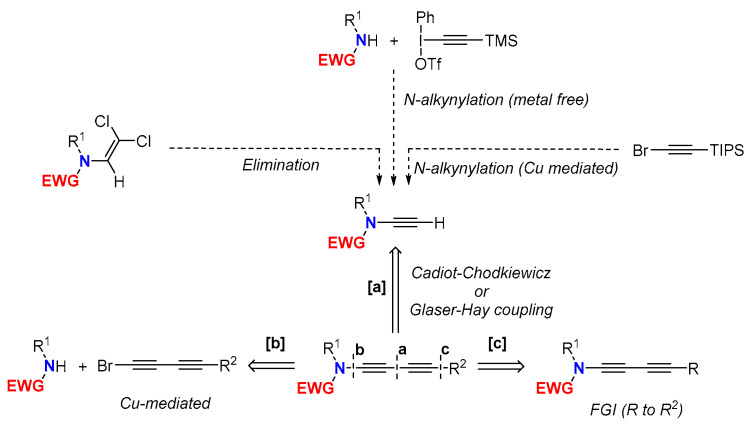
Retrosynthesis of 1,3-butadiynamide.

The majority of described 1,3-butadiynamides are derived from arylsulfonamides (EWG = Toluenesulfonyl (Ts), Methanesulfonyl (Ms), etc.), and only a few examples rely on carbamates (EWG = CO_2_Me) or oxazolidinones. Syntheses of 1,3-butadiynamides follow the basic three retrosynthesis disconnections [**a**–**c**] as shortlisted in Scheme 1.

### 2.1. Disconnection [**a**]: Cu-Catalyzed Cross-Coupling Reactions

Disconnection [**a**] to 1,3-butadiynamides follows common strategies for the assembly of 1,3-bisacetylenes starting from various terminal acetylenes (Glaser–Hay coupling) or cross-couplings of terminal acetylenes with 1-bromoalkynes (Cadiot–Chodkiewicz reaction). The use of terminal ynamides in copper-catalyzed cross-coupling reactions is the major strategy to access both symmetrical and unsymmetrical 1,3-butadiynamides. The required terminal ynamides are readily available by rendering the synthetic strategies outlined in Scheme 1 [13].

**Scheme 2:**
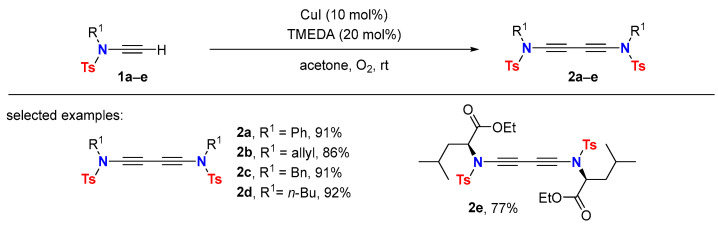
Symmetrical substituted 1,3-diynamides via oxidative Glaser–Hay coupling.

Application of the Glaser–Hay reaction to terminal ynamides proceeds readily affording various sets of symmetrical substituted 1,3-butadiynamides **2a**–**e** (Scheme 2) [22,23].

The oxidative homocoupling of terminal ynamides is usually carried out in acetone at room temperature with CuI-TMEDA (tetramethylethylenediamine) as the catalyst and by exposure to atmospheric oxygen. It is also applicable to chiral amino acid-derived terminal ynamides to deliver 1,3-diynamide **2e** [24].

**Scheme 3:**
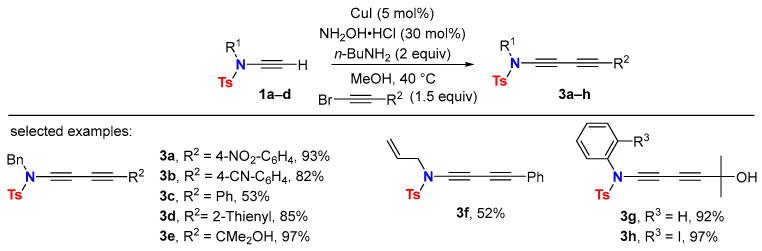
1,3-Butadiynamides via Cadiot–Chodkiewicz cross-coupling reaction.

Unsymmetrical 1,3-butadiynamides **3a**–**h** are available via the Cadiot–Chodkiewicz cross-coupling between terminal ynamides and 1-bromoalkynes (Scheme 3) [25,26]. Reactions are usually performed in methanol at 40 °C and require copper(I) iodide as the catalyst, *n*-butylamine as the base and sub-stoichiometric quantities of hydroxylamine hydrochloride. The use of the latter is crucial to avoid ynamide homocoupling by reducing any copper(II) salts present in the reaction medium. Notably, the complementary approach utilizing bromine- or iodine-terminated ynamides with terminal acetylenes was unsuccessful so far. This is reasoned by the fact that bromine- or iodine-terminated ynamides are difficult to obtain and are quite unstable [27].

**Scheme 4:**
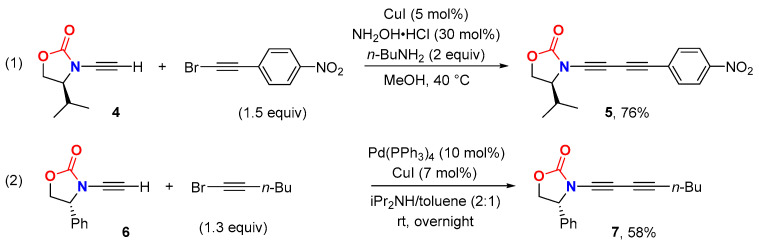
Synthesis of oxazolidine-2-one-derived 1,3-butadiynamides.

The Cadiot–Chodkiewicz reaction with *N*-ethynylated oxazolidine-2-one **4** is also suitable for the synthesis of oxazolidine-2-one-derived 1,3-butadiynamides, which, for example, delivers the chiral push–pull 1,3-butadiynamides **5** in high yields (Scheme 4, (1)) [25].

Furthermore, but limited in scope, the palladium-mediated cross-coupling of ynamide **6** with 1-bromohexyne in (*i*-Pr)_2_NH/toluene (2:1 (*v*/*v*)) gives the corresponding 1,3-butadiynamide **7** via a Sonogashira type reaction (Scheme 4, (2)) [28].

### 2.2. Disconnection [**b**]: Amide N-Alkynylations

**Scheme 5:**
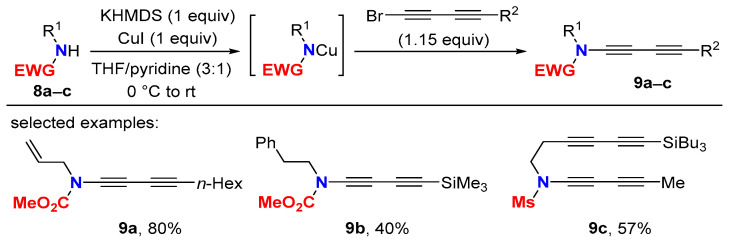
Synthesis of 1,3-butadiynamide via Cu-mediated C-N bond formation.

In analogy to related ynamide approaches, the synthesis of 1,3-butadiynamides via a copper-mediated C-N bond formation between suitable amides and 1-bromo-1,3-butadiynes was described, though only a few examples have been realized so far. Here, the stabilization of the acting copper amide complex prior to the coupling with the 1-bromo-1,3-diyne is mandatory. This is necessary to overpower the quite facile homocoupling of 1-bromodiynes (Scheme 5) [29,30]. C-N bond formation takes place at room temperature, but the drawbacks of requiring stoichiometric amounts of copper iodide and the need of high-quality pyridine should be taken into consideration. Indeed, the use of dry pyridine, freshly distilled over CaH_2_ under inert atmosphere, is required. The reaction conditions are also suitable for and were applied in the synthesis of the *N*-methanesulfonyl 1,3-butadiynamide **9c** (EWG = Ms) [31].

**Scheme 6:**
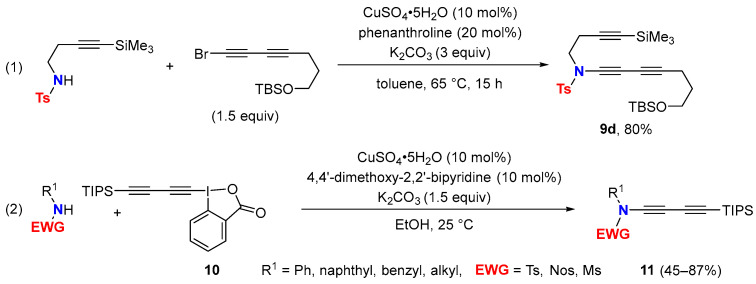
Copper-catalyzed synthesis of 1,3-butadiynamides.

Ynamide syntheses relying on sub-stoichiometric amounts of copper sulfate/1,10-phenanthroline as the catalyst in toluene at 60–95 °C [32] were extended to gain access to 1,3-butadiynamides. Efficiency, however, is lower compared to the protocol leading to carbamate-derived 1,3-butadiyne **9a** via stoichiometric amounts of copper salt (36 vs. 80% yield) [29]. Notably, *N*-alkyl *N*-tosylamides are better substrates, and the synthesis of *N*-tosyl-1,3-butadiynamide **9d** proceeds with high yields under copper-catalyzed cross-coupling conditions (Scheme 6, (1)) [33].

It is worth noting that the synthesis of *N*-aryl *N*-toluenesulfonyl ynamides (R^1^ = Ar) via copper-catalyzed C-N bond-forming protocols with 1-bromo alkynes does not proceed at all, or proceeds in very low yields after several days of heating [34]. Probably, this is due to the weaker nucleophilicity of *N*-aryl *N*-toluenesulfonylamides. Therefore, the Cadiot–Chodkiewicz reaction with terminal ynamides remains the state of the art to obtain 1,3-diynamides having an *N*-aryl group. However, more recently, a copper-catalyzed electrophilic *N*-1,3-diynylation with triisopropylsilyl 1,3-diynyl benziodoxolone (**10**) was described to give straightforward TIPS protected 1,3-butadiynamides **11** including examples with *N*-aryl tosylamides (Scheme 6, (2)) [35].

### 2.3. Disconnection [**c**]: Functional Group Interconversion (FGI) at the C-Terminus

Chemical modifications of ynamides without affecting the triple bond have been scarcely explored until recently [13]. In the case of 1,3-butadiynamides, examples of modifications of the *C*-terminus are even fewer.

**Scheme 7:**
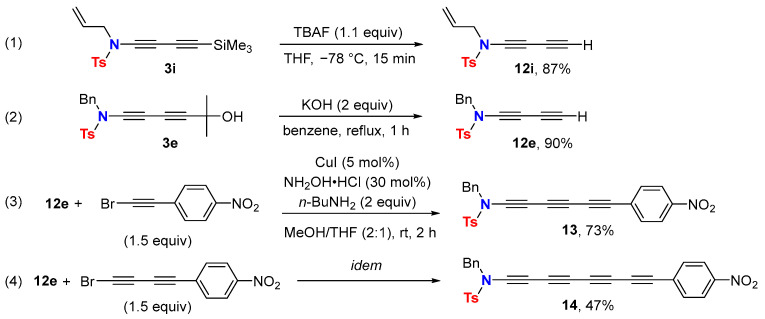
Modification of the 1,3-butadiynamide *C*-terminus.

The synthesis of a terminal 1,3-butadiynamide through desilylation of the corresponding trimethylsilyl protected precursor with tributylammonium fluoride (TBAF) at −78 °C in dry THF proceeds in high yield (Scheme 7, (1)) [36]. Alternatively, access to terminal 1,3-butadiynamides is provided under basic conditions starting from the parent 1,3-butadiynamide terminated by the acetone adduct (Scheme 7, (2)) [25].

1,3-Butadiynamide synthesis followed by transformation to a terminal 1,3-butadiynamide via protective group strategies allows for the development of iterative Cadiot–Chodkiewicz couplings to higher order ethynylogous ynamides. Such iterative cross-couplings offer a straightforward entry to conjugated tri- and tetraynamides **13** and **14** using as coupling partners diynamide **12e** and a 1-bromoalkyne or a 1-bromo-1,3-diyne, respectively (Scheme 7, (3) and (4)) [25].

## 3. Molecular Structure and Electronic Properties

1,3-Butadiynamides and their higher homologues not only gain interest as molecular building blocks in organic synthesis, but they also receive considerable attention for their unique conformation on basis of principal helical twisted molecular orbitals delocalized over the entire conjugated carbon rod, with their optoelectronic properties including NLO performance, and their possible function as molecular wire or as monomer in solid-state topo-chemical polymerizations.

**Figure 2:**
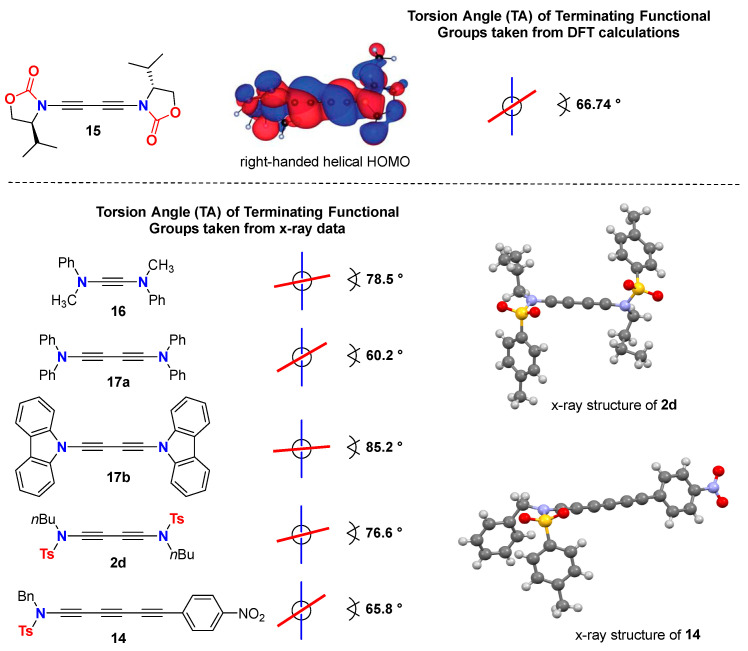
Helical twisted molecular orbitals in extended conjugated oligoynamides.

Whilst axial chirality in certain odd numbered cumulenes like allenes is well-documented, the understanding of chirality based on helical twisted frontier molecular orbitals (Hel-FMO) and its impact on chemical, optical and physical properties is still in its early infancy. Markedly, it is mainly limited to the theoretical and computational description of conjugated cumulenes [37,38], spiroconjugated systems [39] and suitable extended conjugated oligoynes (ECOs) [40,41,42,43]. The latter favor a twisted near orthogonal conformation caused by the combination of repulsive interactions between the terminating functional groups and the constructive orbital overlap upon twisting. Nitrogen atom-terminated oligoynes are predicted of being important members of the ECO family because of both being stable and having helical molecular orbitals delocalized over the entire conjugated polyyne carbon rod (Figure 2).

For example, the high-level density functional theory (DFT) calculation of the still unknown—but synthetically feasible—diynamide **15** predict helical twisted molecular frontier orbitals with axial chiral geometry [41]. For the 1,3-diynamide **15**, a torsion angle (TA) of TA = 66.74° was calculated together with a right-handed helical HOMO (Figure 2). Notably, similar values of TAs can be found in the single crystal structure data of the ynamine **16** (TA = 78.5°) [44], the 1,3-diynamines **17a** (TA = 60.2°) [45] and **17b** (TA = 85.2°) [46], the 1,3-diynamide **2d** (TA = 76.6°) [23] and the 1,3,5-triynamide **14** (TA = 65.8°) [25]. Although these data are taken from solid-state structures, and conformational changes due to crystal structure-packing effects cannot be neglected, all TAs are of similar magnitudes and underline the influence of helical twisted molecular orbitals on the conformational output of 1,3-diynamides and their higher homologues.

**Figure 3:**
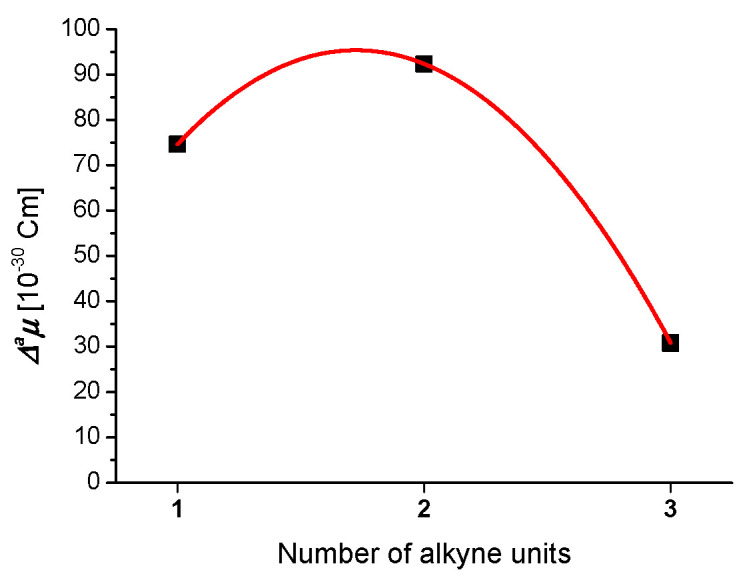
Correlation of the number of alkyne units in ynamides with the magnitude of change of dipole moment from ground to Franck–Condon excited state Δ^a^*μ*.

A set of donor-π-spacer-acceptor “push–pull” ynamides end-capped with nitrogen donor and 4-nitro or 4-cyanophenyl acceptor units was investigated by the electro-optical absorption method (EOAM). The aim of the study was to examine their intramolecular charge transfer (ICT) properties in relation to the extent of the spacing-conjugated oligoyne units [25]. Dipole moments in solution of the ground (*μ*_g_), the Franck–Condon excited state (*μ*_ε_^FC^) and their respective change of dipole moment (Δ^a^*μ*) were obtained. Values of dipole moments for measurements in 1,4-dioxane of selected *para*-nitro-phenyl substituted ynamide derivatives are compiled in Table 1. The observed very high values for the change of dipole moments after transition from the ground to the Franck–Condon excited state show effective ICTs and consequently potential non-linear optical properties for these electronically tunable, extended, conjugated oligoynamides. Notably, the increase of change of dipole moments reaches a maximum for push–pull 1,3-diynamides with two spacing-conjugated alkyne units. Elongation of the conjugated alkyne units from one to two triple bonds results in an increase of the Δ^a^*μ* values, while the ground-state dipole moments *μ*_g_ are almost unaffected. Increasing to three conjugated alkyne units, however, leads to a decrease of Δ^a^*μ* (Figure 3).

Topochemical solid-state polymerization of 1,3-diacetylenes in single crystals gives conjugated polymers that have attracted attention on their physical properties such as conductivity, optical nonlinearity and mechanical strength. Consequently, 1,3-butadiynamides that are often crystalline materials were also investigated in topochemical polymerizations [23,47,48].

## 4. Addition, Cycloaddition and Oxidation Reactions

Non-symmetrical 1,3-butadiynamides—unlike their symmetrical counterparts—often act as internal ynamides. In many examples of addition and cycloaddition reactions, the 1,2-activation of the 1,3-diyne is superior to the 1,4-activation, and the second triple bond remains unaffected in the underlying transformation. However, in view of the richness of acetylene chemistry, the obtained products can often undergo further chemical transformations, broadening the array of available molecular and heterocyclic structures.

### 4.1. Addition Reactions

**Scheme 8:**
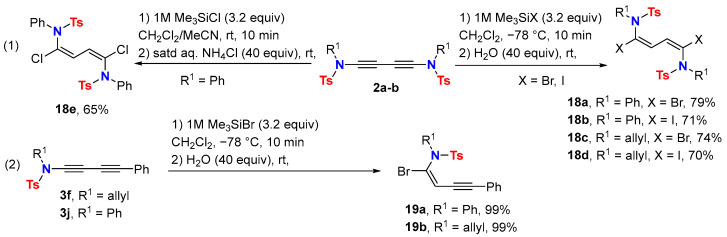
Regio- and stereoselective hydrohalogenation of 1,3-butadiynamide.

Stereodefined 1,3-dienes bearing *N*-substituents are important building blocks in organic synthesis. Simple access to versatile 1,4-dihalogenated *E*,*E*-1,4-dienamides was achieved from readily available symmetrical 1,3-butadiynamides **2a**–**b** in regio- and stereoselective hydrohalogenations (Scheme 8, (1)) [49].

**Scheme 9:**
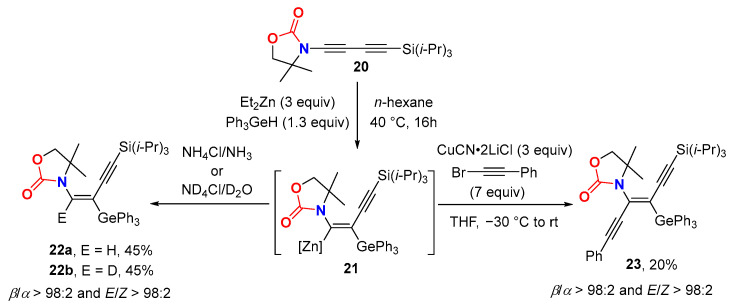
Radical germylzincation towards stereodefined ynenamides.

Hydrogen halides are generated in situ from halotrimethylsilane (Br, I) and water─or chlorotrimethylsilane and saturated ammonium chloride. Under these conditions, the perfect *syn*-addition of H-X (X = Cl, Br) across the ynamide triple bond occurs. The hydrobromination of non-symmetrical 1,3-butadiynamides **3f**,**j** ensues only on the triple bond linked to the nitrogen atom, i.e., the more electron-rich carbon-carbon triple bond, affording the 1-en-3-ynamides **19a**–**b** (Scheme 8, (2)). Notably, the hydrobromination of 1,4-diphenyl-butadiyne does not occur under similar reaction conditions. Germylzincation of 1,3-butadiynamide **20** was achieved with excellent regio- and stereoselectivity using a combination of hydrogermanes and ZnEt_2_ (Scheme 9) [50].

Addition of germanium did not occur in the absence of Et_2_Zn. The proposed mechanism involves the addition of a germanium-centered radical across the triple bond on *β*-position to the *N*-atom in agreement with the underlying polarization of the 1,3-butadiynamide. The resulting α-heteroatom-substituted vinyl radical undergoes ethylzinc group transfer to give the *β*-zincated vinylgermane **21** along with an ethyl radical keeping the radical chain propagation. The (*E*)-form of the vinylgermane radical is favored. Retention of the double bond geometry of the vinylzinc intermediate **21** is observed upon hydrolysis or Cu(I)-mediated C-C bond formation with 1-bromophenylacetylene. The resulting stereodefined tri- and tetrasubstituted ynenamides **22a**–**b** and **23** can be further functionalized through displacement of germanium. Vinylgermanes gain increasing attention as alternatives to vinylsilanes and vinylstannanes for the preparation of stereodefined alkenes because of their low toxicity, increased stability towards protonolysis and facile transformation into vinyl halides.

**Scheme 10:**
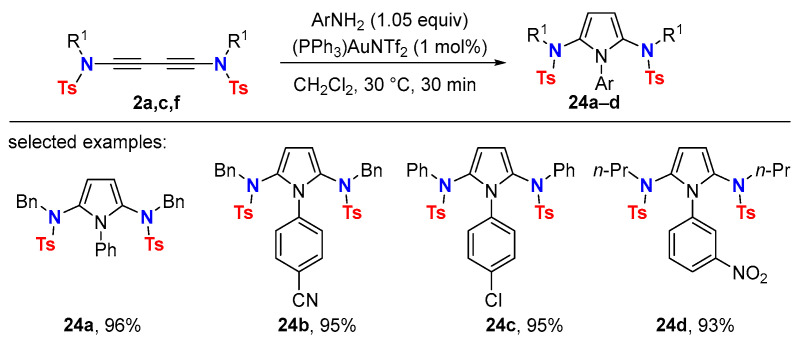
Gold-catalyzed synthesis of 2,5-diamidopyrroles.

Gold-catalyzed double hydroaminations of symmetrical substituted 1,3-butadiynamides **2a**,**c**,**f** with anilines readily proceed at room temperature and deliver the corresponding 2,5-diamido-*N*-arylpyrroles **24a**–**d** (Scheme 10) [51].

**Scheme 11:**
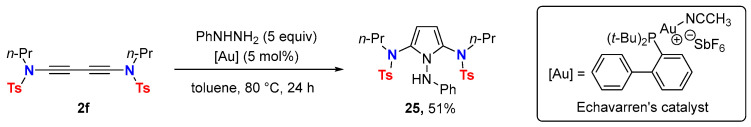
Gold-catalyzed synthesis of a 1,2,5-trisubstituted pyrrole with phenylhydrazine.

Moreover, the 1,2,5-trisubstituted pyrrole **25** is accessible by using phenylhydrazine as the hydroamination reagent. In this case, thermal activation is required, as well as the use of the more reactive Echavarren’s gold catalyst derived from the bulky and electron-rich di-*t*-butylphosphinobiphenyl ligand (Scheme 11) [51].

**Scheme 12:**
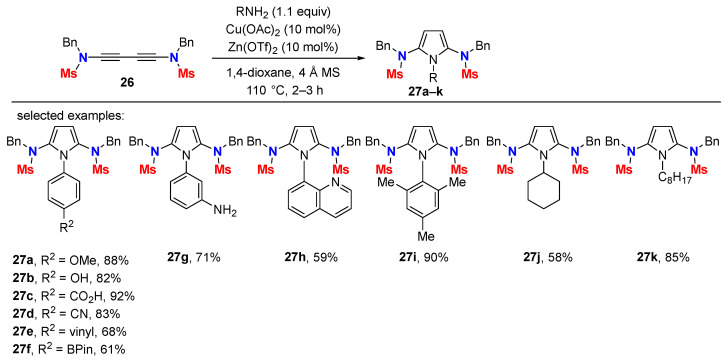
Copper-catalyzed synthesis of 2,5-diamidopyrroles.

Copper catalysis in the presence of a zinc additive is also effective in the pyrrole series and proceeds with excellent functional group tolerance [52]. In comparison to the gold-catalyzed protocol, the scope of 2,5-diamidopyrroles available from 1,3-butadiynamides is considerably broadened as primary aliphatic amines, and sterically hindered anilines are now suitable hydroamination reagents (Scheme 12).

**Scheme 13:**
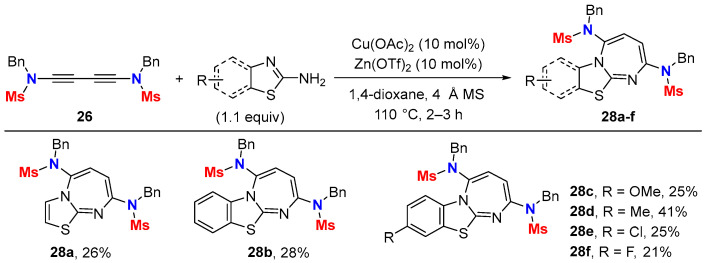
Copper-catalyzed synthesis of diazepines derived from 1,3-diynamides.

Interestingly, the copper-catalyzed reaction of 1,3-butadiynamide **26** with 2-amino (benzo)thiazoles as nucleophiles does not deliver pyrrole derivatives. Now, fused diazepines **28a**–**f** are formed via a formal [4+3] annulation sequence (Scheme 13) [52].

**Scheme 14:**
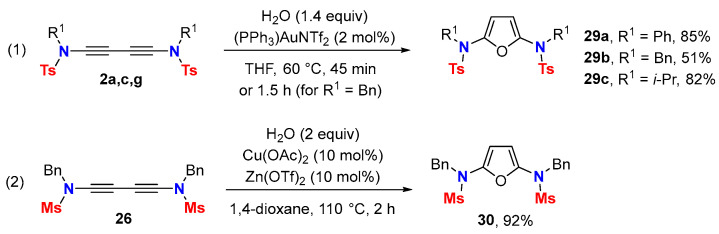
Gold- or copper-catalyzed synthesis of 2,5-diamidofurans.

Symmetrical substituted 1,3-butadiynamides also serve as substrates in gold catalyzed hydration leading to the 2,5-diamidofurans **29a**–**c** (Scheme 14, (1)) [51]. Interestingly, the dimethanesulfonyl analogue of **29b**—the 2,5-diamidofuran **30**—was obtained in a better yield using a copper(II) catalyst in the presence of a zinc additive (Scheme 14, (2)) [52].

**Scheme 15:**
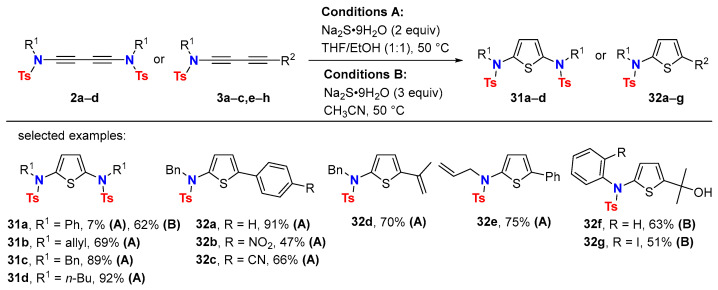
Metal-catalyst-free synthesis of 2-amido- and 2,5-diamidothiophenes.

The metal-catalyst-free synthesis of 2,5-diamido-thiophenes **31a**–**d** using 1,3-butadiynamides **2a**–**d** and Na_2_S•9H_2_O as the sulfur-providing source proceeds under very mild conditions (Scheme 15, Conditions A) [26]. Both symmetrical as well as the non-symmetrical 1,3-diynamides undergo a formal 1,4-functionalization of the parent 1,3-diyne unit. In the case of diynamide **2a** (R^1^ = Ph), whose electrophilicity is enhanced by conjugation of the ynamide unit with the *N*-phenyl group, these reaction conditions were not suitable. This is probably because of side reactions resulting from the addition of ethanol across the triple bond. Switching to acetonitrile as the solvent was the key for success (Conditions B). Now, the corresponding thiophene **31a** is obtained in 62% yield. Importantly, the method is likewise effective with unsymmetrical 1,3-butadiynamides **3a**–**c**,**e**–**h** giving a straightforward entry to variously functionalized 2-amidothiophenes **32a**–**g** (Scheme 15).

**Scheme 16:**
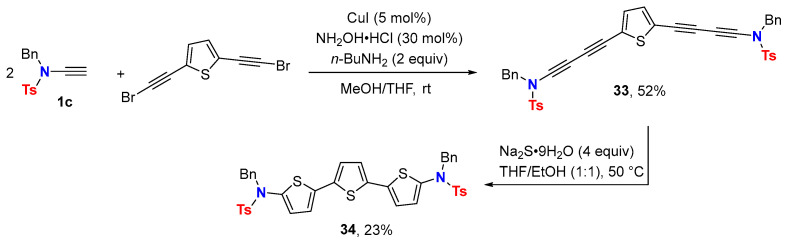
Synthesis of a terthiophene.

This new approach to 2-amido- or 2,5-diamidothiophenes is extendable to the synthesis of terthiophenes (Scheme 16). The required tetrayne **33** bearing two linked 1,3-diynamide units is accessible via a double Cadiot–Chodkiewicz coupling between a terminal ynamide and bis(bromoalkynyl)thiophene. Application of the developed reaction conditions to substrate **33** delivers the diamido-capped terthiophene **34** having a string of *N*,*S* heteroatoms embedded in a highly π-conjugated molecular scaffold. Electron-rich terthiophenes are interesting as active materials in organic electronics such as organic transistors or organic photovoltaic cells.

### 4.2. Cycloaddition Reactions

**Scheme 17:**
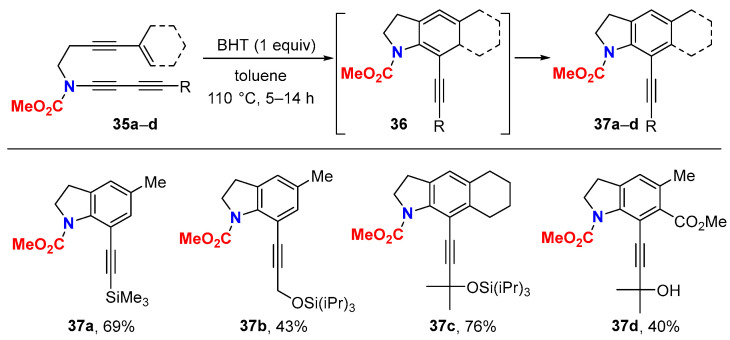
Intramolecular [4+2] cycloaddition with 1,3-butadiynamides to give 7-alkynyl indolines via DHDA reaction.

Intramolecular [4+2] cycloadditions of 1,3-butadiynamides **35a**–**d** tethered to an enyne moiety furnish functionalized 7-alkynyl indolines **37a**–**d** (Scheme 17) [53]. Yields improve in the presence of BHT, which not only suppresses the polymerization of enyne substrates, but also facilitates the isomerization of the cyclic allene intermediate **36** into indoline **37** via proton/hydrogen atom transfer. As alkyl-substituted ynamides with only one C–C triple bond give low yields in related [4+2] cycloadditions, the hydrogenation of the triple bond of indolines **37a**–**d** is an alternative and offers a valuable entry to the parent alkyl-substituted indolines. In these Didehydro–Diels–Alder (DHDA) reactions the 1,3-butadiynamide serves as the formal dienophile. Notably, this has to be distinguished from its use in Hexa-Dehydro-Diels–Alder (HDDA) reactions that will be discussed in Chapter 6, where the 1,3-butadiynamide unit functions as the formal 4π cycloaddition partner.

**Scheme 18:**
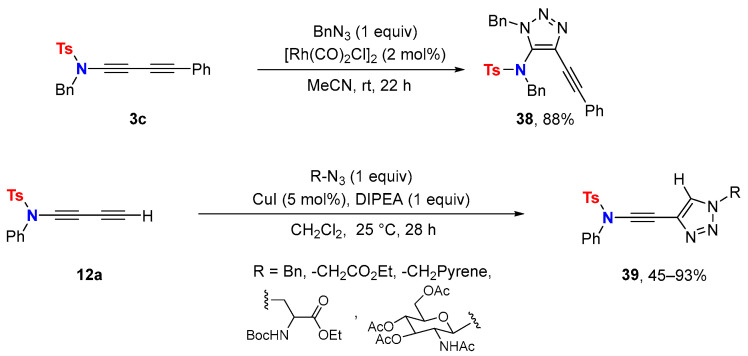
Intermolecular azide-alkyne [3+2] cycloaddition with 1,3-butadiynamides to give 4-alkynyl triazoles.

Azide-alkyne [3+2] cycloadditions of 1,3-butadiynamide **3c** using rhodium catalysis afford 4-alkynyl 5-amino-triazole **38** [54], whereas the copper-catalyzed addition of azides to terminal 1,3-butadiynamides provide alternative ynamide-derived azide-alkyne click-products **39** (Scheme 18) [35]. Click reactions with 1,3-diynamides are exclusively chemo- and regioselective with stoichiometric amounts of azides. The exclusion of air and moisture is unnecessary in the case of the rhodium catalysis, whereas high yields in copper-mediated azide-alkyne [3+2] cycloadditions are obtained only under strict anhydrous conditions.

**Scheme 19:**
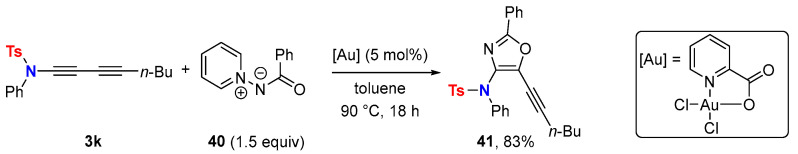
Intermolecular [3+2] cycloaddition of aminide **40** to 1,3-butadiynamide **3k** to give 5-alkynyloxazole **41**.

The gold-catalyzed cycloaddition of 1,3-butadiynamide **3k** with aminide **40** as *N*-acyl nitrene equivalent gives 5-alkynyloxazole **41** as a single regioisomer in 83% yield (Scheme 19) [55]. This formal [3+2] cycloaddition is based on the use of the robust and air-stable dichloro(pyridine-2-carboxylato)gold(III) complex as a pre-catalyst.

### 4.3. Oxidation Reactions

**Scheme 20:**
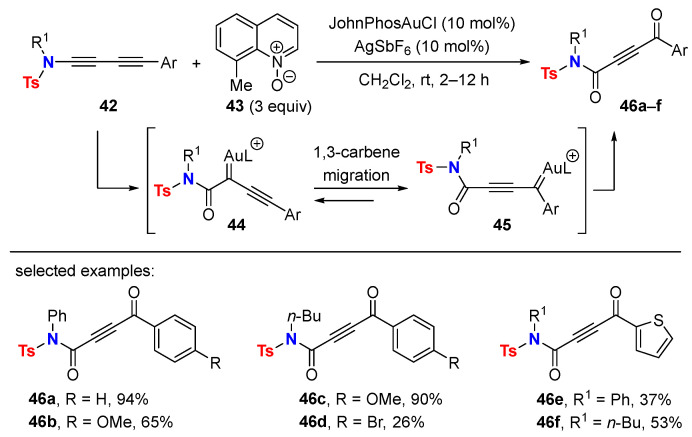
Au(I)-catalyzed 1,4-oxidation of 1,3-butadiynamides.

The catalytic oxidation of aryl 1,3-butadiynamides **42** with 8-methylquinoline oxide (**43**) using a cationic gold catalyst in the presence of a silver salt delivers the 1,4-oxidation products **46** (Scheme 20) [56].

In agreement with other gold-catalyzed oxidations of ynamides with pyridine *N*-oxides, a striking chemoselectivity for *C*(1) oxidations is observed leading to the α-carbonyl gold carbene intermediate **44**, which, in the case of the ethynylogous ynamide, undergoes a 1,3-carbene migration affording intermediate **45** [57]. The 1,3-migration tautomer **45** is then further oxidized to give the double oxidation products **46**. Finally, this oxidative process results in a 1,4-functionalization of the 1,3-diynamide moiety.

**Scheme 21:**
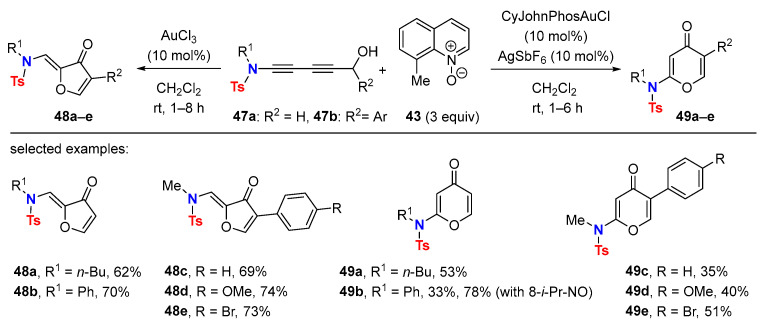
Au(I)- and Au(III)-mediated oxidative cyclization of 5-hydroxy-1,3-butadiynamides.

Interestingly, a different chemical outcome is observed with 5-hydroxy-1,3-butadiynamides **47a**–**b**, which refrain from *C*(1) oxidation. Here, an atypical *C*(3) oxidation and a further gold-mediated oxidative cyclization lead to either furan-3-ones **48a**–**e** or to pyran-4-ones **49a**–**e** with AuCl_3_ and CyJohnPhosAuCl/AgSbF_6_, respectively (Scheme 21) [56]. The formation of pyran-4-ones **49** from the less basic *N*-phenyl *N*-toluenesulfonylamide-derived substrates **47** (R^1^ = Ph) is more efficient with 8-*iso*-propylquinoline oxide, which is illustrated in the synthesis of product **49b** with 78 vs. 33% yield. Importantly no exchange between the two cyclic ketones occurs in the presence of the gold catalysts.

**Scheme 22:**
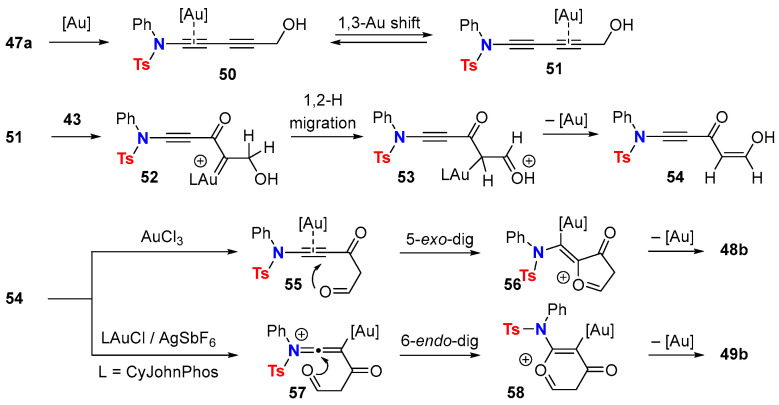
Proposed mechanism for the Au-catalyzed oxidative formation of cyclic ketones.

DFT calculations support a mechanism for the chemoselective conversion of **47a** (R^1^ = Ph, R^2^ = H) into cyclic ketones **48b** or **49b** (Scheme 22).

The gold π-complexed alkynes **50** and **51** in Scheme 22 are interconvertible. The 1,4-functionalization through the oxidation at *C*(3) relies on an energetically favored complex **51** in comparison to **50** because the energy barrier of the oxidation of **51** with 8-methylquinoline oxide (**43**) is calculated as lower. The resulting α-oxo gold carbene **52** undergoes 1,2-hydride migration leading to intermediate **53**, which evolves to the 3-oxo-5-enol intermediate **54** by releasing a gold species. Complexation of the triple bond of **54** with less alkynophilic (more Lewis acidic) Au(III)Cl_3_ induces an intramolecular carbonyl addition to the alkyne moiety via a 5-exo-dig cyclization. The latter affords intermediate **56**, whereas the formation of a keteniminium species **57** in the case of the cationic gold catalyst facilitates 6-*endo*-dig cyclization to give intermediate **58**. A mechanism for the transformation of the 1,3-diynamide **47b** bearing an *aryl*-substituted hydroxy moiety was unfortunately not provided by the authors. The structure of the corresponding products **48c**–**e** and **49c**–**e**, however, suggests that a less often observed 1,2-aryl migration takes place [58]. This might also explain the net result with substrate **47** bearing an *alkyl*-substituted hydroxy moiety. The migratory aptitude of an alkyl group to an electron deficient center typically follows the order H > Ph > alkyl.

## 5. Metal-Catalyzed Cascade-Type Cyclization and Annulation Reactions

### 5.1. Intramolecular Processes

The activation of 1,3-butadiynamides with transition metal π-acids to form keteniminium ions provide superb opportunities of cascade-type cyclization and annulation reactions. For intramolecular processes, only gold catalysts have been used so far [59]. The involved gold catalysts often vary according to the 1,3-butadiynamides or to the reaction conditions applied.

**Scheme 23:**
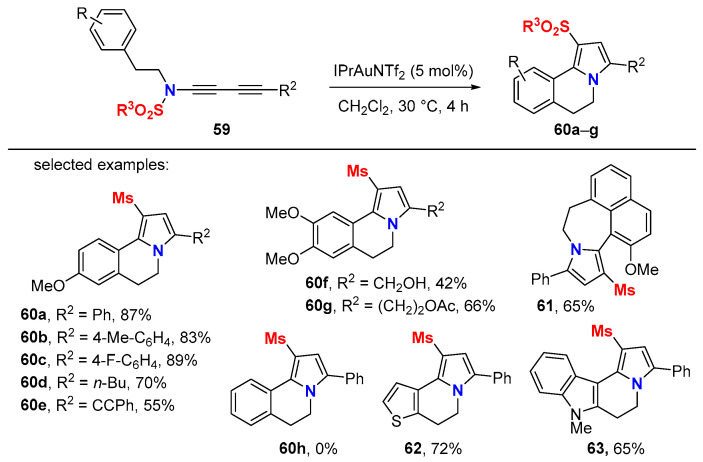
Gold-catalyzed synthesis of sulfone-containing pyrrolo[2,1-*a*]tetrahydroisoquinolines.

1,3-Butadiynamides **59** that are linked via the ynamide nitrogen by a two carbon tether to electron-rich benzenes or electron excess heteroaromatics readily undergo a gold-catalyzed cascade reaction with IPrAuNTf_2_ (IPr = 1,3-bis(diisopropylphenyl)imidazole-2-ylidene), leading to sulfone-containing pyrrolo[2,1-*a*]tetrahydroisoquinolines **60a**–**g** (Scheme 23) [60]. The cationic nature of the gold catalyst is crucial. The counter anion NTf_2_^−^ acts as a proton transfer shuttle and facilitates the overall process, which notably involves a formal 1,4-sulfonyl migration. No reaction takes place in the absence of an electron-donating group on the phenyl ring (see **60h**). This stands for the electrophilic aromatic substitution step within the reaction cascade. Further functional groups susceptible to react with the gold catalyst, such as alkyne, hydroxy or acetate groups, are tolerated (see **60e**–**g**). Remarkably, other structural motifs are also accessible through this process, such as the pyrrolo-azepine **61** or the heterocyclic systems **62** and **63**.

**Scheme 24:**
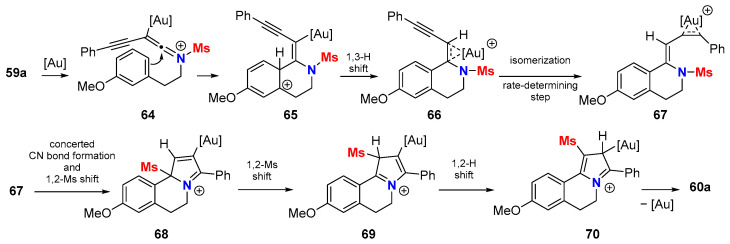
Proposed mechanism for the gold-catalyzed transformation of 1,3-butadiynamides into sulfone-containing pyrrolo[2,1-*a*]tetrahydroisoquinolines.

The main intermediates involved in this cascade reaction find support from DFT calculations (Scheme 24). The gold keteniminium species **64** resulting from a regioselective attack of the electrophilic gold catalyst to the 1,3-diynamide moiety undergoes an intramolecular arylation (Vilsmeier–Haack-type reaction) followed by re-aromatization through a 1,3-H shift mediated by the counter anion NTf_2_^−^ to deliver intermediate **66**. The isomerization of *cis*-**66** to *trans*-**67** required for the second annulation process is the proposed rate-determining step. This second cyclization takes place via a concerted C-N bond formation and includes a 1,2-methanesulfonyl-(Ms) migration to give intermediate **68**. A second 1,2-Ms shift followed by a 1,2-H shift, which is greatly facilitated by the counter anion NTf_2_^−^, delivers intermediate **70**, which finally furnishes product **60a** after demetallation.

**Scheme 25:**
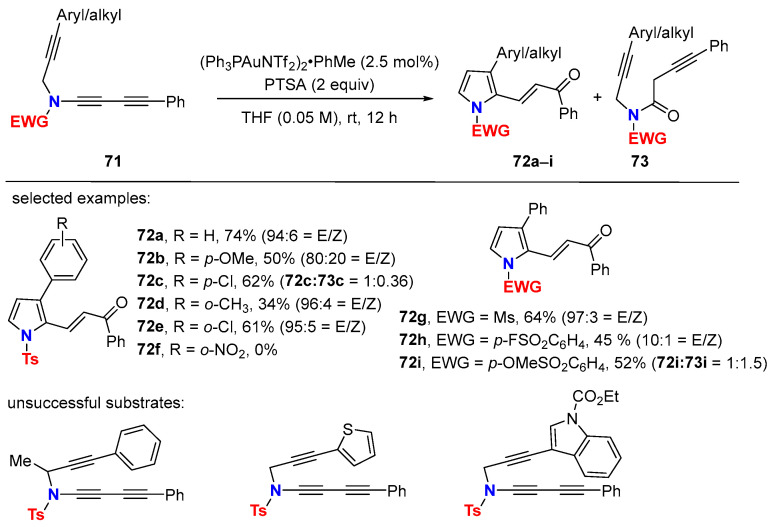
Gold(I)-catalyzed *para*-toluenesulfonic acid (PTSA) promoted the cycloisomerization of 1,3-diynamides to α,β-unsaturated ketones.

A related gold(I)-catalyzed *para*-toluenesulfonic acid (PTSA) promoted cycloisomerization of 1,3-diynamides **71** gives access to α,β-unsaturated ketones **72** together with minor amounts of the 1,3-diynamide hydration product **73** (Scheme 25) [61]. The studied transformation was limited to 1,3-diynamides terminated with a phenyl group, and the other substitution pattern was incompatible mismatched with the desired reactivity.

**Scheme 26:**
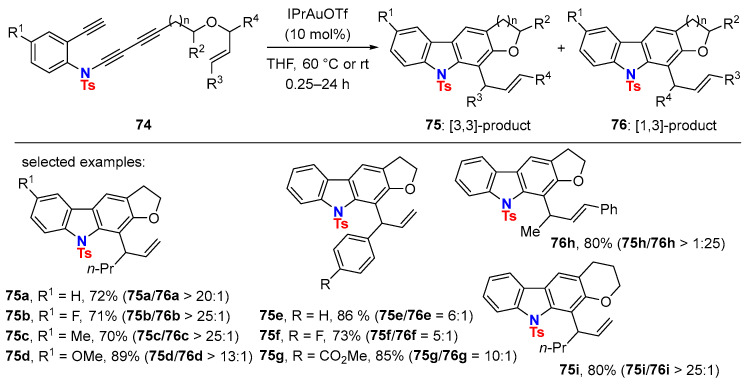
Gold-catalyzed formal HDDA/carboalkoxylation reaction cascade.

*C*-terminal-functionalized 1,3-butadiynamides **74** linked to a terminal alkyne and an allylic ether were used in a reaction cascade involving a dual gold-catalyzed process. The reaction cascade includes an intramolecular carboalkoxylation and a subsequent charge-accelerated [3,3] sigmatropic rearrangement to deliver carbazoles **75** as the major products (Scheme 26) [62]. Compound **76**, resulting from a [1,3] sigmatropic rearrangement, was formed as the minor side product in most cases. However, reversal of regioselectivity took place when the latter process was electronically favored (see **76h**).

**Scheme 27:**
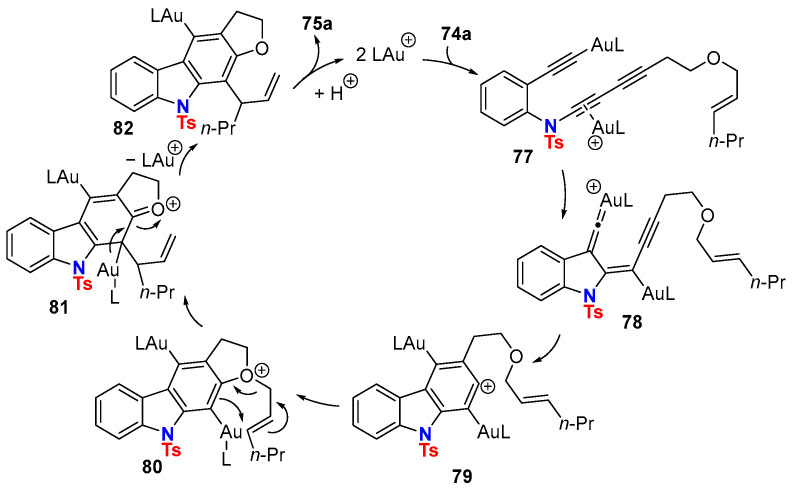
Proposed mechanism for the Au-catalyzed carboalkoxylation reaction cascade.

Triynes bearing an internal instead of a terminal alkyne moiety are unsuitable for this reaction cascade in agreement with the proposed mechanism that is being initiated by a σ,π dual activation of triyne **74a** to give intermediate **77** (Scheme 27). The latter undergoes a 5-*exo*-dig cyclization to give the gold vinylidene carbenoid **78**, which then cyclizes to furnish the *ortho*-Au phenyl cation **79**. The gold-complexed aryne **79** is trapped by the ether group leading to the oxonium species **80**. A charge-accelerated [3,3] sigmatropic rearrangement of **80** gives intermediate **81** followed by re-aromatization delivering **82** along the release of the gold ion. The final step of the proposed mechanism is the protodeauration of **82** to furnish product **75a** by releasing the active gold catalyst.

### 5.2. Intermolecular Processes

**Scheme 28:**
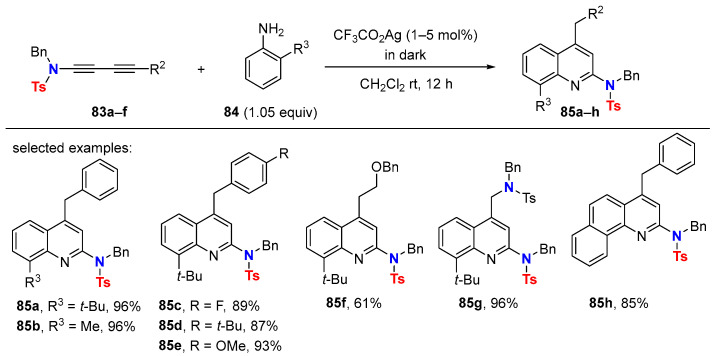
Ag(I)-catalyzed synthesis of 2-amidoquinolines.

As highlighted in chapter 4, the metal-catalyzed double hydroaminations of symmetrical 1,3-butadiynamides with anilines gives 2,5-diamido-*N*-arylpyrroles. Surprisingly, in related transformations, *ortho*-substituted anilines as hydroamination reagent trigger a cationic driven reaction cascade after the first hydroamination step. For example, silver(I)-catalyzed transformations of 1,3-butadiynamides **83a**–**f** with *ortho*-substituted anilines **84** bearing electron-donating substituents give 2-amidoquinolines **85a**–**h** (Scheme 28) [63]. The combination of steric and electronic factors is crucial to gain preference for the formation of quinoline over pyrrole-derived products. One purpose of the electron-donating group in *ortho* position of the aniline is to facilitate the sequential electrophilic aromatic substitution step that rationalizes product formation. Symmetrical substituted 1,3-butadiynamides are also suitable substrates as shown in the example of bis-amido quinoline 85g (96% yield).

**Scheme 29:**
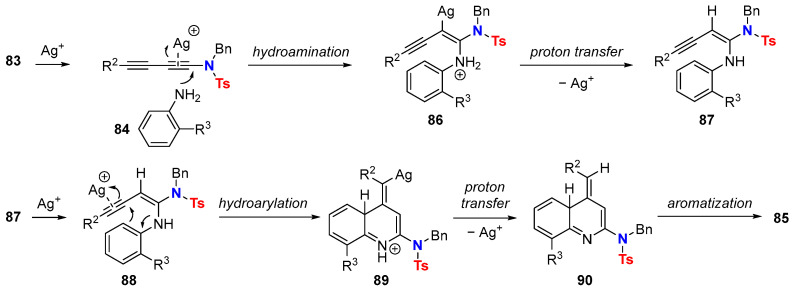
Proposed mechanism for the Ag(I)-catalyzed synthesis of 2-amidoquinolines.

The proposed mechanism is depicted in Scheme 29. Silver-catalyzed intermolecular hydroamination of 1,3-butadiynamide **83** with aniline **84** delivers the enyne intermediate **86**, which, after proton transfer and protodeargentation, leads to **87**. Silver complexation of the remaining triple bond in **87** facilitates intramolecular hydroarylation via an electrophilic aromatic substitution process to give **89**. Subsequent proton transfer and re-aromatization results in the final quinoline **85**.

**Scheme 30:**
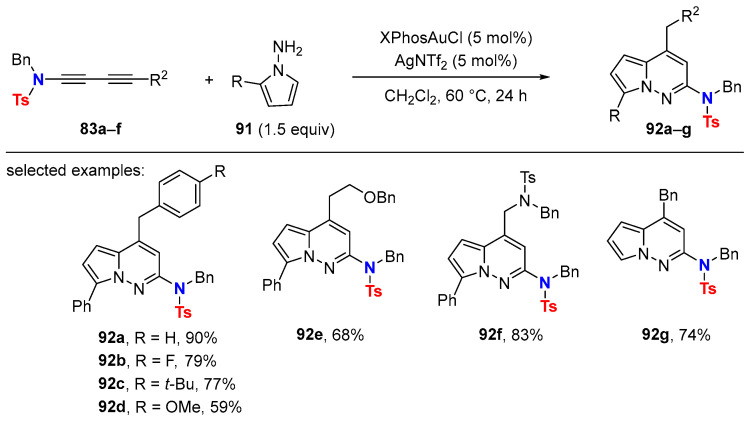
Au(I)-catalyzed synthesis of 2-aminopyrrolo[1,2-b]pyridazines.

The synthesis of 2-aminopyrrolo[1,2-b]pyridazines **92a**–**g** from non-symmetrical 1,3-butadiynamides **83a**–**f** and 1-aminopyroles **91** via a related strategy is also reported (Scheme 30) [64]. The overall reaction cascade proceeds through Au(I)/Ag(I)-mediated C-N/C-C bond formations to end up with readily substituted 2-aminopyrrolo[1,2-b]pyridazines **92**.

**Scheme 31:**
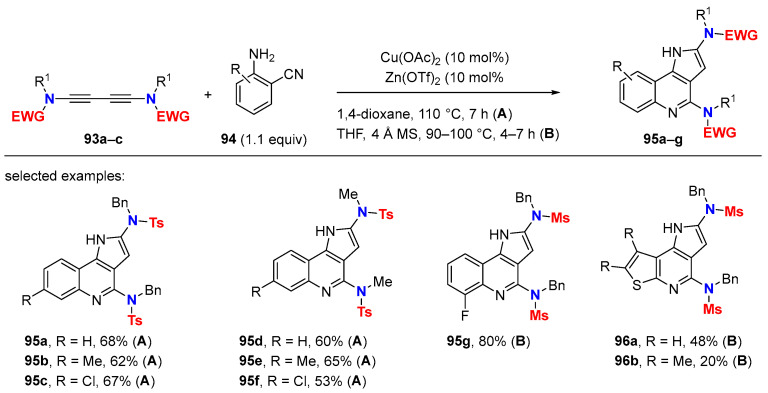
Cu(II)/Zn(II)-catalyzed synthesis of 1*H*-pyrrolo[3,2-c]quinolines.

The reaction of symmetrical substituted 1,3-butadiynamides **93a**–**c** with *ortho*-cyano anilines **94** provides aryl-fused 1*H*-pyrrolo[3,2-c]quinolines **95a**–**g** within a single step via a dual catalyst process (Scheme 31) [65]. Heteroaryl-fused 1*H*-pyrrolo[3,2-c]quinolines **96a**–**b** are also accessible using 2-amino-3-cyano thiophenes as nucleophiles. Both catalysts—copper(II) acetate and zinc(II) triflate—are necessary for the cascade reaction to be effective. Finally, the overall sequence realizes the formation of one C-C and two new C-N bonds.

**Scheme 32:**
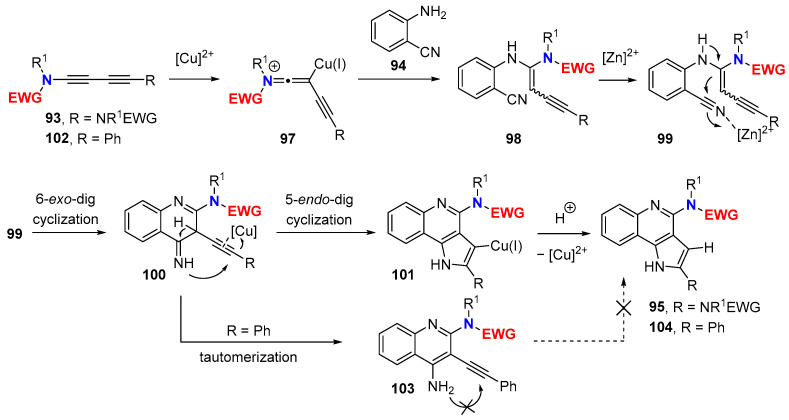
Proposed mechanism of Cu(II)/Zn(II)-catalyzed synthesis of 1*H*-pyrrolo[3,2-c]quinolines.

Activation of 1,3-butadiynamides via metal–π coordination of a π-acidic copper(II)-species to form the metal keteniminium **97** is the initiating step of the proposed mechanism (Scheme 32). This facilitates the intermolecular hydroamination to give enamine **98** followed by activation of the cyano group in **98** through coordination with the Zn catalyst. The resulting Zn-complexed **99** can undergo a 6-*exo*-dig cyclization via intramolecular nucleophilic attack of the enamine moiety producing **100**. Activation of the remaining carbon–carbon triple bond by Cu(II) facilitates a 5-*endo*-dig cyclization providing **101**, which upon protodemetallation leads to the 1*H*-pyrrolo [3,2-c]quinoline product **95**. In the case of non-symmetrical 1,3-butadiynamides **102** (R = Ph), tautomerization of intermediate **100** leads to the monocyclization product **103**, which is a major side product in the reaction. Quinoline **103** is unable to provide **104** through a second annulation sequence, as shown in a separate control experiment. Therefore, an equimolar mixture of products **103** and **104** resulting from both pathways is observed starting from 1,3-butadiynamide **102** even after prolongation of the reaction time. Notably, diphenyl-1,3-diacetylene did not undergo any reaction under these reaction conditions. This one more time underlines the differences in reactivity between 1,3-diynamides and other 1,3-butadiynes.

**Scheme 33:**
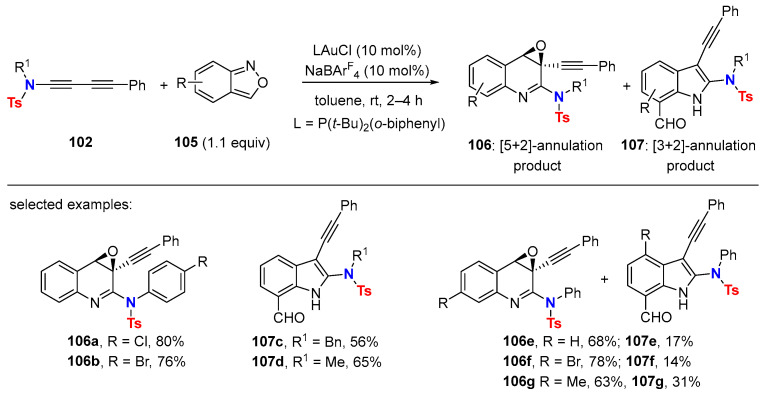
Gold-catalyzed reaction between 1,3-butadiynamides and anthranils.

The outcome of gold-catalyzed reactions of 1,3-butadiynamides **102** with anthranils **105** is substrate-dependent, preferentially affording the formal [5+2] annulation products **106** in the case of 1,3-butadiynamides bearing an electron-deficient *N*-aryl group, while in contrast, [3+2] annulation products **107** are preferentially formed with more electron-rich *N*-alkyl 1,3-butadiynamides (Scheme 33) [66].

**Scheme 34:**
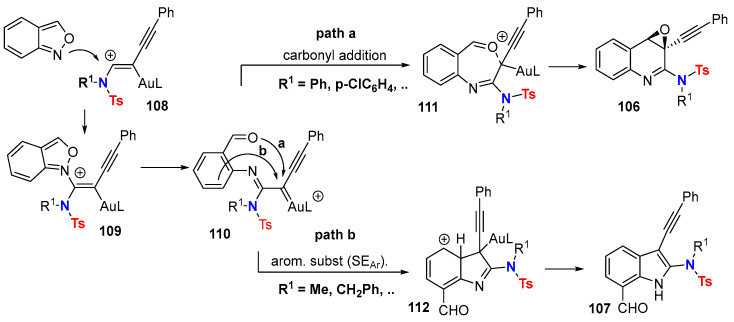
Proposed mechanism for Au(I)-catalyzed reaction of 1,3-butadiynamides with anthranil.

DFT calculations rationalize the observed chemoselectivity. Gold activation of the 1,3-butadiynamide followed by *N*-attack of anthranils leads to intermediate **109**, which evolves into the energetically more favorable α-imino gold carbene **110** (Scheme 34). The latter undergoes carbonyl addition to give **111** bearing a seven-membered cycle (**path a**). Although intermediate **111** is higher in energy than intermediate **112**, resulting from carbene arylation (**path b**), the free energy barrier for the formation of **111** is smaller than the one of **112**. Moreover, the conversion of **111** into quinoline oxide **106** is highly exothermic and expected to be almost barrierless. The preference for **path a** relies on the presence of the alkynyl substituent, which brings favorable steric and electronic effects to facilitate the formation of seven-membered ring intermediate **111**. These results are specific to 1,3-butadiynamides [67].

Gold-catalyzed oxidative cascade reactions of (het)aryl-tethered 1,3-butadiynamides, using pyridine *N*-oxide as the oxidant, encompass efficient protocols to access complex polycyclic heteroaromatics within a single step. Here, the active gold species is an α-carbonyl gold carbene, whose obviously faster reaction with the proximal electron-rich aromatic group overrides the otherwise typical 1,4-dicarbonyl formation (Section 4.3, Scheme 20).

**Scheme 35:**
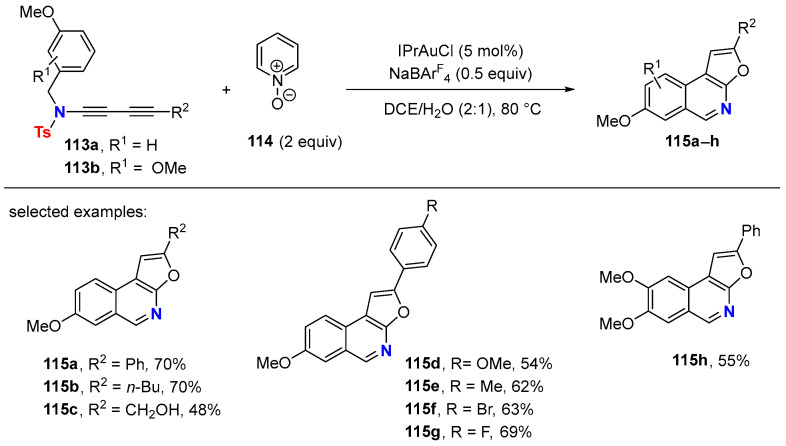
Au(I)-catalyzed synthesis of furo[2,3-*c*]isoquinolines.

**Scheme 36:**
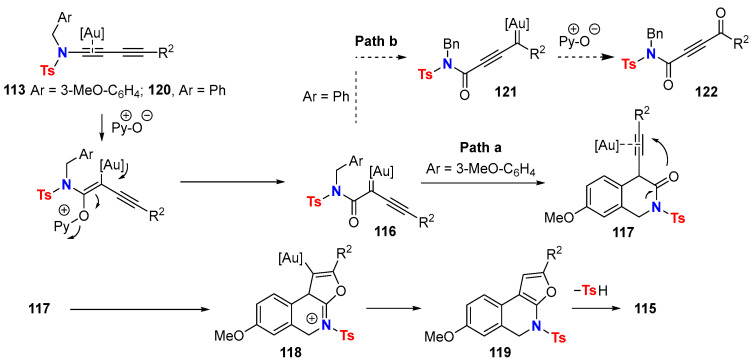
Proposed mechanism for the synthesis of furo[2,3-*c*]isoquinolines.

The reaction of 3-methoxyphenyl-tethered 1,3-butadiynamides **113a**–**b** with pyridine *N*-oxide (**114**), using catalytic amounts of IPrAuCl in the presence of NaBAr^F^_4_, leads to furo [2,3-*c*]isoquinolines **115a**–**h** (Scheme 35) [68]. The nature of the substituent at the terminal 1,3-diynamide end—alkyl or phenyl—for the outcome of the reaction cascade is negligible, but the presence of the 3-methoxy group on the tethered aryl ring is mandatory for the reaction cascade to occur. In the absence of the 3-methoxy group, in the case of substrates *N*-benzyl 1,3-butadiynamide **120** (Ar = Ph, Scheme 36), the double oxidation product **122** is the only product formed under the reaction conditions (**Path b**, Scheme 36).

According to the proposed mechanism, the gold-activated 1,3-butadiynamide **113** is oxidized by pyridine *N*-oxide (**114**) to give α-carbonyl gold carbenoid **116**, which is in situ trapped by the aryl group through CH insertion delivering intermediate **117** (**path a**, Scheme 36). Carbonyl addition on the activated second triple bond of **117** leads to the fused tricyclic intermediate **118**, which undergoes protodeauration to **119**. The transformation of intermediate **119** into the final polycyclic heteroaromatic product **115** via release of sulfonic acid is highly favorable and might be the driving force of the total transformation according to the authors. The involvement of **119a** (R^2^ = Ph) was evidenced by conducting the reaction at room temperature that provided **119a** (25% isolated yield) along with unreacted 1,3-butadiynamide **113a**. Full conversion of **119a** into product **115a** was achieved upon heating at 80 °C.

**Scheme 37:**
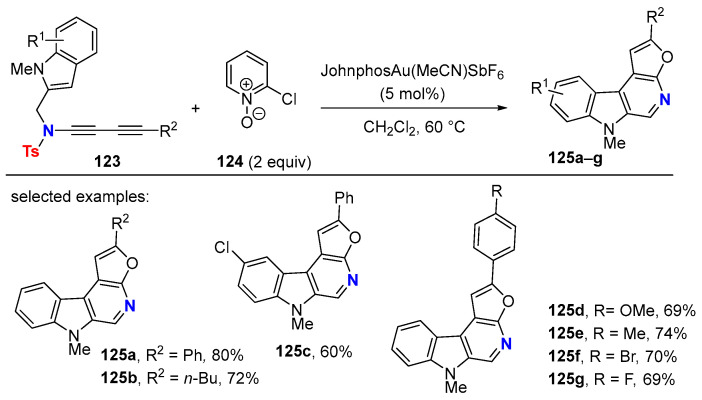
Gold-catalyzed synthesis of 6*H*-furo[3′,2′:5,6]pyrido[3,4-*b*]indoles.

Accordingly, as extension, the assembly of fused tetracyclic heteroaromatics **125a**–**g** was achieved via the same strategy using *N*-methyl indolyl-tethered 1,3-butadiynamides **123** as the substrates and 2-chloropyridine *N*-oxide (**124**) as the oxidant (Scheme 37).

**Scheme 38:**
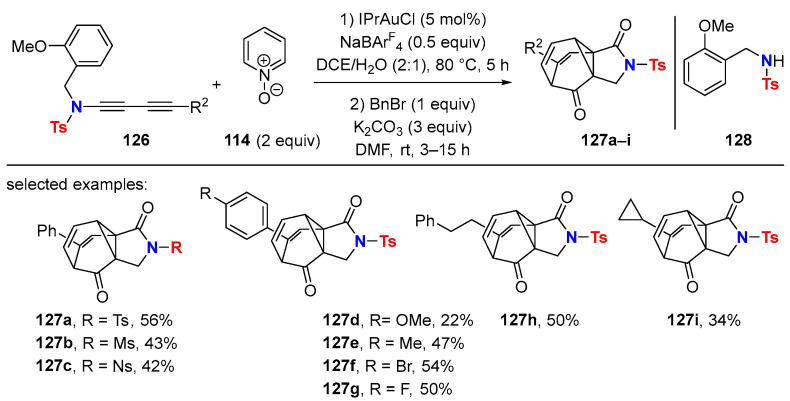
Gold-catalyzed synthesis of polycyclic product **127**.

Strikingly, the outcome of the gold-catalyzed oxidative cascade cyclization of methoxyphenyl-tethered 1,3-butadiynamides is tunable by modifying the position of the methoxy group [69]. Indeed, the gold-catalyzed reaction of 2-methoxyphenyl-tethered 1,3-butadiynamides **126** with pyridine *N*-oxide (**114**) selectively delivers the polycyclic compounds **127a**–**i** with a barbalan-type carbon skeleton along with the byproduct **128**, resulting from hydrolysis and saponification of **126** (Scheme 38).

**Scheme 39:**
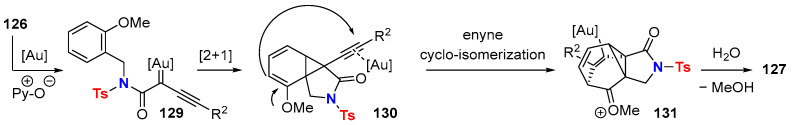
Proposed mechanism for the synthesis of **127**.

Arene CH insertion from in situ generated α-carbonyl gold carbenoids is not involved here in comparison to the case of the parent α-carbonyl gold carbenoid **116** depicted in Scheme 36. The preferential mechanistic path now is the intramolecular cyclopropanation of the arene unit by the carbenoid **129** to give norcaradiene **130** (Scheme 39). [3,3] Sigmatropic enyne cycloisomerization between the vinyl methoxy ether and the gold-activated alkynyl moiety leads to the formation of oxocarbenium ion **131**, which after hydrolysis delivers the polycycle **127**.

**Scheme 40:**
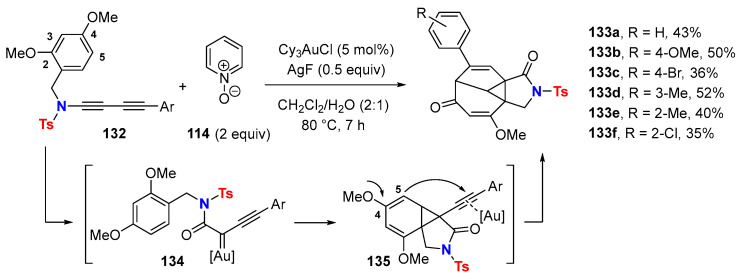
Au(I)-catalyzed reaction of 2,4-dimethoxyphenyl-tethered 1,3-butadiynamide **132** with pyridine *N*-oxide (**114**).

A different set of polycycles now having an eight-membered ring moiety is available through a related gold-catalyzed dearomatization/cycloisomerization process by placing an additional methoxy group and varying the substitution pattern of dimethoxyphenyl-tethered 1,3-butadiynamides substrates. For example, 2,4-dimethoxyphenyl-tethered 1,3-butadiynamides **132** are selectively converted into the polycyclic products **133a**–**f** upon reaction with pyridine *N*-oxide (**114**) using Cy_3_AuCl/AgF as the catalyst (Scheme 40). In this case, the 2-methoxy group remains intact, whereas the additional 4-methoxy group is involved in the formal [3,3] sigmatropic enyne rearrangement.

**Scheme 41:**
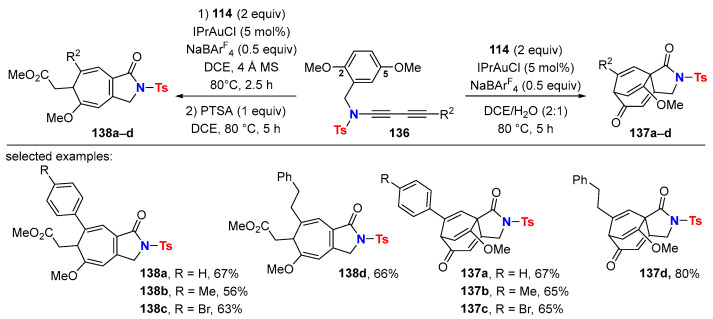
Au(I)-catalyzed reaction of 2,5-dimethoxyphenyl-tethered 1,3-butadiynamides **136** with pyridine *N*-oxide (**114**).

Using 2,5-dimethoxyphenyl-tethered 1,3-butadiynamides **136** as substrates makes the two fused *N*-heterocyclic structures **137a**–**d** and **138a**–**d** available, depending on whether the reaction is carried out in the presence of water or under anhydrous conditions, respectively (Scheme 41).

**Scheme 42:**
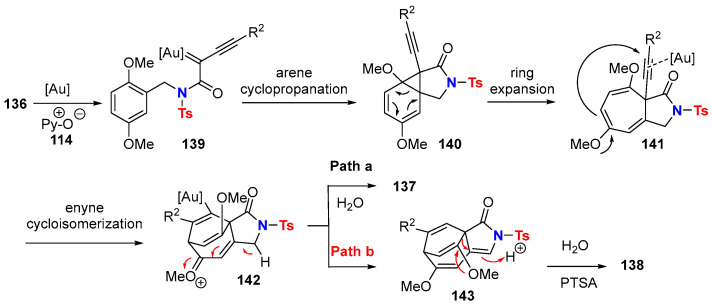
Proposed mechanism for the Au(I)-catalyzed reaction of 2,5-dimethoxyphenyl-tethered 1,3-butadiynamides **136** with pyridine *N*-oxide (**114**).

The proposed mechanism involves the generation of a reactive α-carbonyl gold carbene species **139**, which undergoes a formal [2+1] cycloaddition with the adjacent arene moiety to give norcaradiene intermediate **140** (Scheme 42). The methoxy-substituted cyclopropane unit facilitates norcaradiene to cycloheptatriene ring expansion, leading to **141**. Subsequent intramolecular enyne cycloisomerization delivers intermediate **142**, whose hydrolysis furnishes the tricyclic products **137**. Under anhydrous conditions, intermediate **142** converts into product **143** via deprotonation and protodeauration. Acidic treatment of **143** induces the cleavage of one carbocycle, delivering the bicyclic compounds **138**. Importantly, such a pathway is supported by the isolation of compound **143a** (R^2^ = Ph) and its conversion into the corresponding product **138a** by treatment with PTSA.

Whereas the majority of the so far reviewed transformations of 1,3-butadiynamides rely on activation of one or two of its carbon–carbon triple bonds by either Brønsted or Lewis acids, or by transition metal catalysts acting both as π-acid and Lewis acid, or Au catalysts transforming 1,3-diynamides into metal carbenoid intermediates, other principles of ynamide activation might result from transforming 1,3-butadiynamides into [4]cumulenimines or related highly unsaturated π-conjugated molecular scaffolds.

**Scheme 43:**
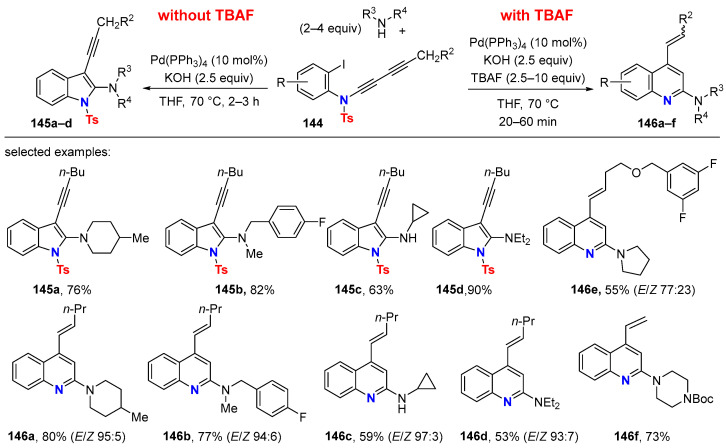
Divergent Pd-catalyzed reaction cascades towards indole and quinoline motifs.

Recently, divergent palladium-catalyzed reaction cascades for the selective synthesis of either 2-amino-3-alkynyl-indoles **145a**–**d** or 2-amino-4-alkenylquinolines **146a**–**f** from 1,3-butadiynamides **144** and primary or secondary amines were established (Scheme 43) [70]. Starting from identical or similar substrates, the outcome of the reaction giving either indole or quinoline motifs is switchable, respectively, by the absence of presence of TBAF.

**Scheme 44:**
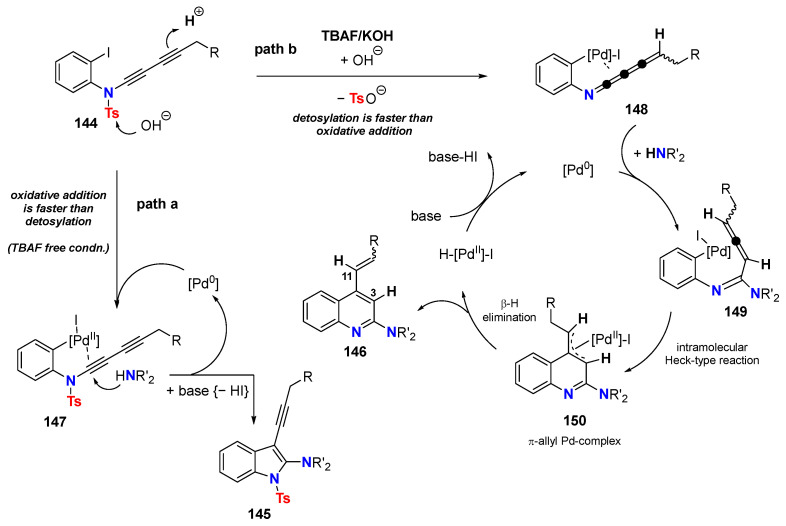
Proposed mechanism for the Pd-catalyzed divergent synthesis of either 2-amino-3-alkynyl-indoles (**path a**) or 2-amino-4-alkenylquinolines via [4]cumulenimine intermediate (**path b**).

Under similar reaction conditions than those applied for the synthesis of 2-aminoindoles through Pd-catalyzed heteroannulation reaction from *N*-alkynyl-2-haloanilides [71], the 1,3-butadiynamides **144** behave like internal ynamides. The second triple bond remains unaffected through the transformation, whose key intermediate is the σ,π-chelated palladium species **147** (Scheme 44, **path a**)**.** The latter results from the initial oxidative addition of the Pd(0) catalyst to the iodophenyl moiety of 1,3-butadiynamides **144**. Metal–π complexation of the ynamide triple bond in **147** facilitates intermolecular amine addition delivering the 3-alkynyl indoles **145** selectively. Strikingly, in the presence of TBAF, both triple bonds are engaged to convert 1,3-butadiynamides **144** in 2-aminoquinolines **146a**–**f** in one pot (Scheme 44, **path b**). The combination TBAF/KOH is believed to induce cleavage of the tosyl group either as the first step or after the oxidative addition of the palladium catalyst to the iodophenyl moiety. Now, the key intermediate is the palladium-chelated [4]cumulenimine **148**. The intermolecular amine addition to **148** selectively occurs at the α-carbon delivering the allene-derived intermediate **149**. Subsequent intramolecular Heck-type reaction furnishes the annulated π-allyl palladium species **150**, which undergoes *β*-hydrogen elimination to produce 4-alkenyl quinolines **146**.

The [4]cumulenimine structure in **148** is unprecedented. However, the deuterium labeling experiment shows the selective deuterium incorporation into the C3 and C11 positions of the quinoline products **146** in agreement with the formation of the transient species **148**.

## 6. HDDA Cascade Reactions

The hexa-dehydro-Diels–Alder (HDDA) reaction of 1,3-diynamides tethered to 1,3-butadiynes delivers highly reactive arynes (dehydrobenzenes) after a formal intramolecular [4π + 2π] cycloaddition process [17]. They are subsequently trapped by in situ intra- or inter-molecular additions to the dehydrobenzene carbon–carbon triple bond, leading to a variety of new heterocyclic structures and useful products. The HDDA reaction itself was discovered in 1997 [72,73] and further developed. However, since 2012, it has received considerable attention, and the underlying process was coined with the term HDDA [18]. The field of HDDA reactions is rapidly expanding, including substrate variations and development of new aryne-trapping modes. The latter takes vast advantage of the fact that typical reagents necessary for generating arynes are absent and therefore cannot interfere [19,20,21]. Studies have shown that reagent-free HDDA reactions proceed through a stepwise mechanism via diradical intermediates. Significant acceleration of the process is observed when the formal 2π cycloaddition partner in the underlying [4π + 2π] cycloaddition process bears an alkynyl substituent (1,3-butadiynyl unit) [33,74]. This is why tetraynes rather than triynes are frequently used in HDDA cascade reactions.

**Scheme 45:**
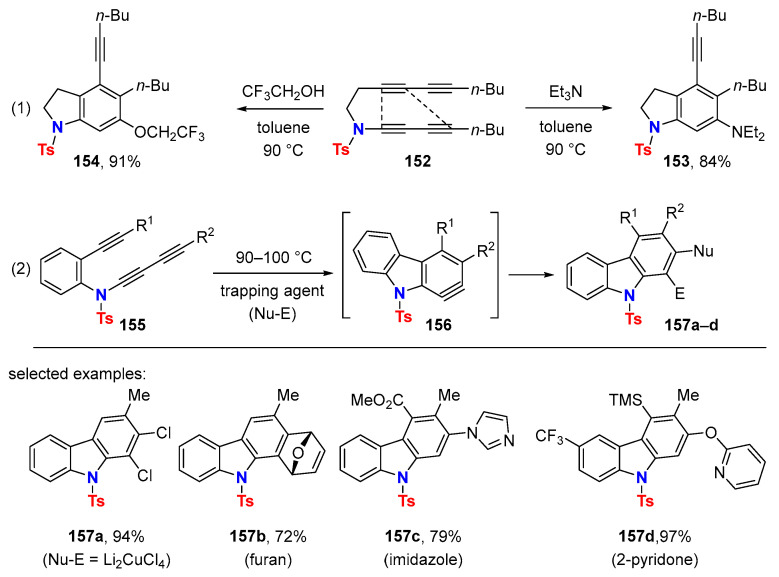
Regioselective “trapping reactions” of 1,3-butadiynamide-derived arynes.

1,3-Butadiynamides tethered to monoalkynes or 1,3-butadiyne moieties are privileged substrates for HDDA reactions because aryne formation proceeds chemo- and regioselectively due to the intrinsic polarization of the 1,3-diynamide unit. Notably, intramolecular HDDA reactions with 1,3-diynamides belong to transformations relying on a 1,4-functionalization of 1,3-diynamides, although the underlying process is step-wise. The follow-up reaction of those 1,3-diynamide-originated arynes continues being highly regioselective, i.e., for the addition of nucleophiles to the aryne. For example, the thermal reaction of tetrayne **152** with triethylamine or trifluoroethanol regioselectively furnishes indolines **153** and **154**, respectively (Scheme 45, (1)) [75]. A novel entry to functionalized carbazoles **157a**–**d** from triyne substrates **155** is accessible through perfectly regioselective nucleophilic addition to HDDA-generated carbazolynes **156** (Scheme 45, (2)) [76].

**Scheme 46:**
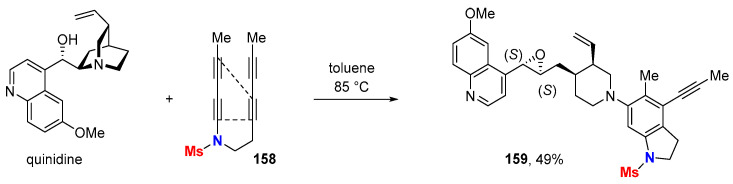
Reaction of a HDDA-generated aryne with quinidine.

The discriminating reactivity of HDDA-generated arynes from 1,3-butadiynamides was further highlighted by chemo- and regioselective transformations using structurally complex multifunctionalized “aryne-trapping agents” taken from the nature pool. For example, the reaction of tetrayne **158** with the cinchona alkaloid quinidine, which displays several potential reacting sites and/or reacting modes, delivers the single indoline product **159** (Scheme 46) [77].

**Scheme 47:**
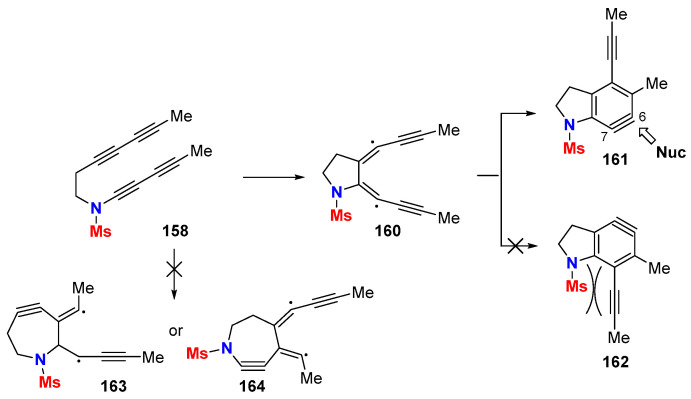
Mechanistic pathway of the formation of a single HDDA-generated aryne from 1,3-butadiynamide-derived substrates.

Experimental results of intramolecular HDDA reactions with 1,3-diynamide were rationalized by DFT calculations (Scheme 47) [78]. Amongst possible diradical intermediates formed in the principal first step of the HDDA reaction of tetrayne **158**, the aza-cycloheptynes **163** and **164** can be ruled out, because they are higher in energy by 35.9 and 41.7 kcal/mol, respectively, compared to the pyrrolidine species **160**. This largely originates from the twofold propynyl stabilization inherent in biradical **160** and the high triple bond strain energy in arynes **163** and **164**. The formation of intermediate **160** is the rate-determining step. Steric factors govern the selective formation of aryne **161** in the second step. The activation barrier to aryne **161** and **162** is 4.3 and 6.6 kcal/mol, respectively, and relates to differences in product stability. Aryne **162** is less stable than **161** by 3.4 kcal/mol because of the steric hindrance between the methanesulfonyl group and the propynyl moiety.

Computational studies, moreover, explain why nucleophilic additions to indoline aryne **161** take place selectively on the C-6 rather than the C-7 position (Scheme 47) [79]. Transition state distortion energies determine regioselectivity. In other words, the aryne **161** displays unsymmetrical bending distortion, and the nucleophilic addition occurs at its flatter and more electropositive end.

**Scheme 48:**
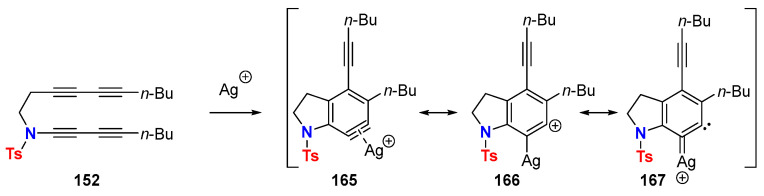
Ag(I)-complexed aryne species in silver-catalyzed HDDA reactions.

The silver-catalyzed HDDA reaction is an interesting alternative to the sole thermal version, eventually affording different reaction outcomes [80]. Ag(I) complexation of the aryne allows for its stabilization while retaining high reactivity. The reactive intermediate resulting from the interaction of aryne with silver salts is described as the silver-bound aryl cations **165**, **166** or by its mesomeric 1,2-carbene-silver carbenoid **167** (Scheme 48).

### 6.1. Sole Thermally Induced HDDA Cascade Reactions

**Scheme 49:**
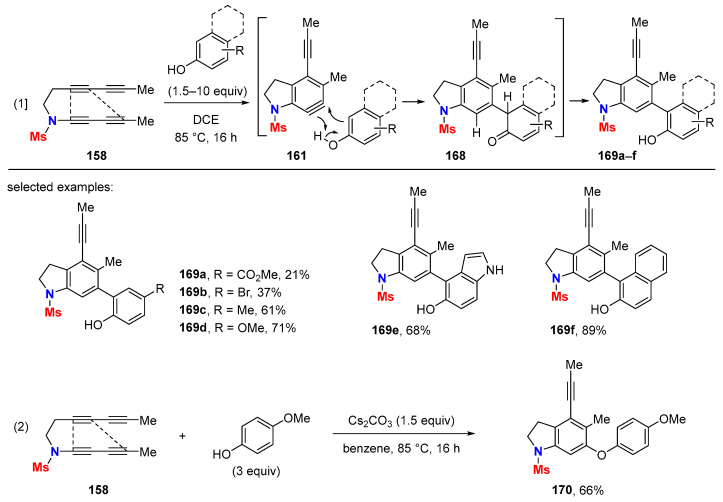
Biaryl vs. diaryl ether synthesis through aryne reactions with phenols.

*Aryne trapping* via *C-C bond formation*: The HDDA-generated benzyne **161** reacts selectively with phenols and delivers 2-hydroxybiaryl compounds **169a**–**f**, and not the expected diaryl ethers, which are archetypal products of arynes generated by elimination reactions (Scheme 49, (1)) [81]. Indeed, sole thermal HDDA reactions proceed under neutral conditions, without the presence of any base or metal reagent, in contrast to classical methods of benzyne generation. The biaryl junction occurs at the *ortho*-position to the hydroxyl group via a “concerted phenol-ene-type” reaction. On the other hand, in the presence of cesium carbonate, the thermal reaction of tetrayne **158** with 4-methoxyphenol gives diaryl ether **170** as a single product (Scheme 49, (2)) [81].

**Scheme 50:**
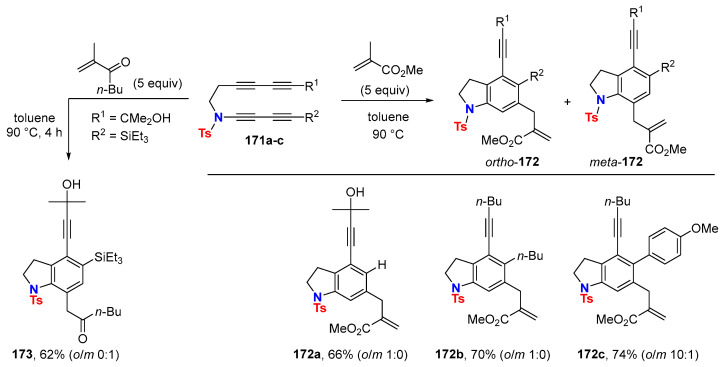
Intermolecular Alder-ene reactions with 1,3-butadiynamide-derived arynes.

Inter- and intramolecular Alder-ene reactions with HDDA-generated arynes lead to a variety of differently substituted indolines. The reaction between 1,3-butadiynamides **171a**–**c** with methyl methacrylate regioselectively delivers the *ortho*-isomers of indolines *ortho-***172a**–**c** (Scheme 50) [82].

However, the steric bulk of the R^2^ substituent has an influence on the Alder-ene reaction outcome. The conversion of 1,3-butadiynamide **171c** substituted with a bulky silyl group (R^2^ = SiEt_3_) preferentially gives indoline *meta*-**173** with 2-methyl-hepten-3-one.

**Scheme 51:**
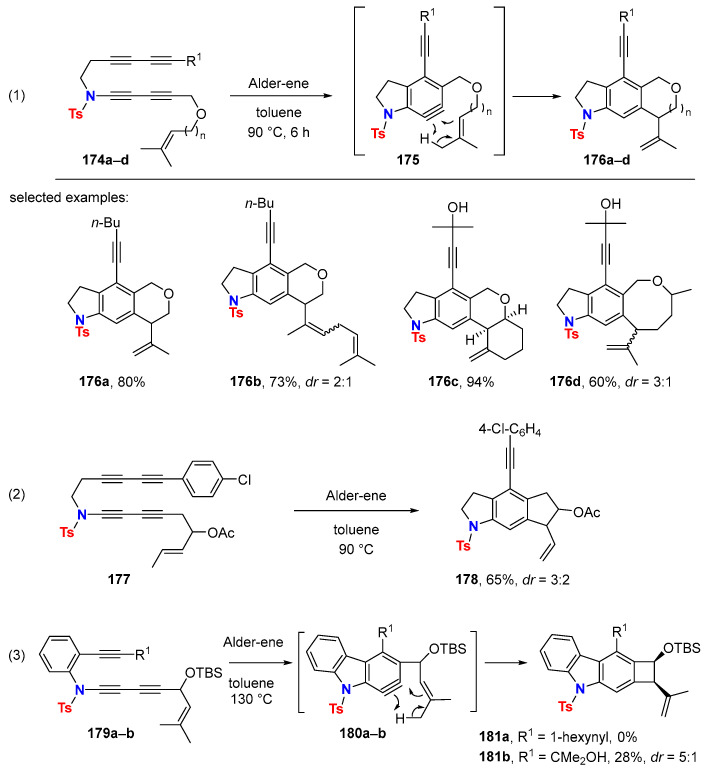
Alder-ene reactions of 1,3-butadiynamide-(**179a**–**b**)-derived arynes **180a**–**b**.

Intramolecular HDDA reactions followed by intramolecular Alder-ene sequences were realized with 1,3-diynamides **174a**–**d** to deliver the linear annulated indolines **176a**–**d** (Scheme 51, (1)) [83]. The chemoselective formation of aryne **175** makes the Alder-ene reaction the most favorable pathway amongst all other possible aryne reacting modes. Extending the tether from three to four atoms delivers eight-membered ring products like compound **176d**. Tetrayne **177** bearing a shorter two-atom tether with a methyl-substituted alkene also undergoes an Alder-ene reaction to deliver tricyclic compound **178** (Scheme 51, (2)) [84]. Isoprenyl-tethered tetrayne **179a** displaying an even shorter one-atom link fails to produce the corresponding Alder-ene product **181a** (R^1^ = 1-hexynyl) even under more drastic conditions [85]. However, increase of steric pressure with the rather bulky isopropanol group in triyne **179b** (R^1^ = C(Me)_2_OH) facilitates the formation of the corresponding benzocyclobutene **181b** (Scheme 51, (3)).

**Scheme 52:**
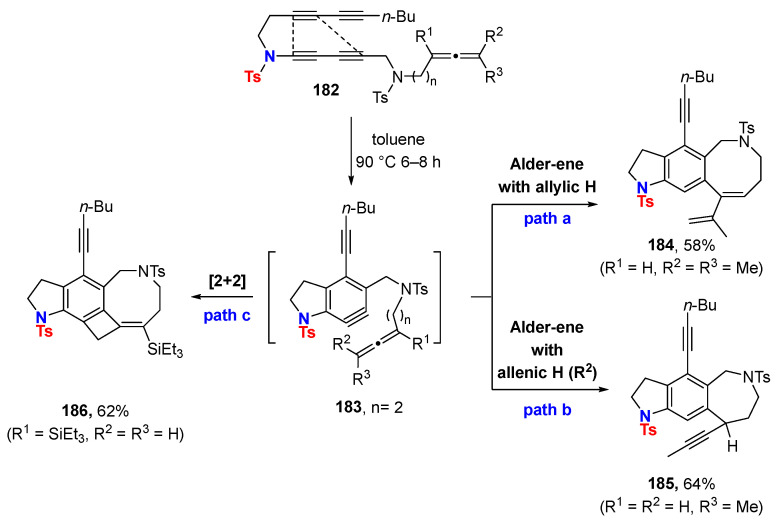
Intramolecular Alder-ene reactions or [2+2] cycloadditions with ynamide-derived tetraynes tethered with an allene.

Thermal HDDA reactions of allene-tethered 1,3-butadiynamides **182** deliver the corresponding aryne intermediates **183**, whose reactivity and selectivity are divergent. The outcome of the intramolecular reaction of the allene with the aryne moiety depends on the substitution pattern of the allene and on the length of the tether (Scheme 52) [86]. The Alder-ene reaction involving an allylic C-H bond is favored with trisubstituted allenes (**path a**) leading to the eight-membered ring containing product **184**. In contrast, the allenic C-H bond is preferentially involved with mono- and 1,3-disubstituted allenes affording the seven-membered Alder-ene product **185** (**path b**). Finally, [2+2] cycloaddition is the preferred path of intramolecular aryne reactions with a 1,1-disubstituted terminal allene furnishing the tetracyclic compound **186** (**path c**).

**Scheme 53:**
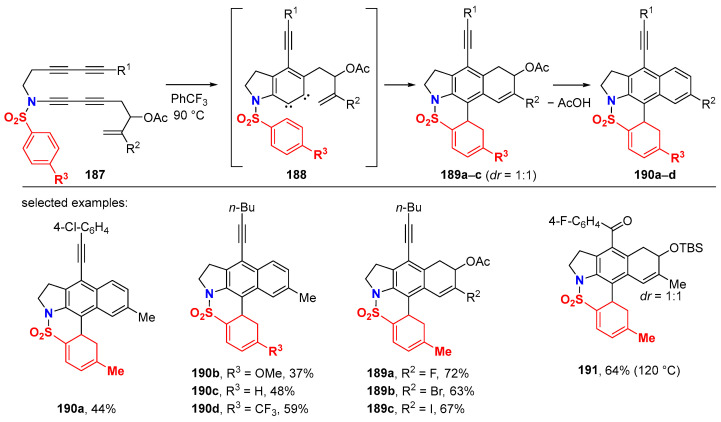
Synthesis of pentacyclic products via HDDA-aryne-mediated arene dearomatization reaction.

The sole thermal reaction of *N*-arenesulfonyl 1,3-butadiynamides **187**, spacing a terminal alkene moiety by a two-atom tether, leads to the pentacyclic structures **189** or **190** via an unprecedented aryne-mediated dearomatization of the phenyl connected to the sulfonyl moiety (Scheme 53) [84]. This transformation reveals the 1,2-dicarbene character of HDDA-generated arynes (intermediate **188**). The efficiency of the reaction increases with cation-stabilizing substituents on the alkene (R^2^) and electron-withdrawing substituents on the arenesulfonyl group (R^3^). Interestingly, compounds **189a**–**c**, bearing a haloalkene moiety (R^2^ = halogen), do not undergo subsequent acetic acid elimination to give the corresponding aromatization products **190**. It is also worth noting that a ketone-containing triyne is a suitable substrate delivering the corresponding pentacyclic product **191** at a slightly higher reaction temperature of 120 °C.

**Scheme 54:**
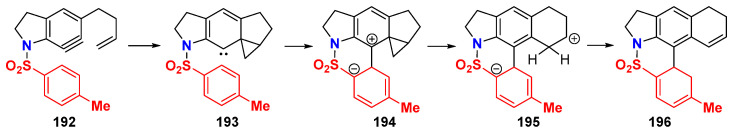
Main intermediates of the proposed mechanistic pathway.

A plausible mechanism, underlined by DFT calculations carried out on the simplified aryne structure **192**, is given in Scheme 54. Alkene cyclopropanation is feasible due to the 1,2-dicarbene character of the HDDA-generated aryne and gives carbene **193**. The latter undergoes nucleophilic addition onto the electron-deficient phenylsulfonyl moiety delivering 1,3-zwittterion **194**. Subsequent ring expansion of the cyclopropane generates the 1,6-zwitterion **195**, which leads to the final product **196** via intramolecular proton transfer.

**Scheme 55:**
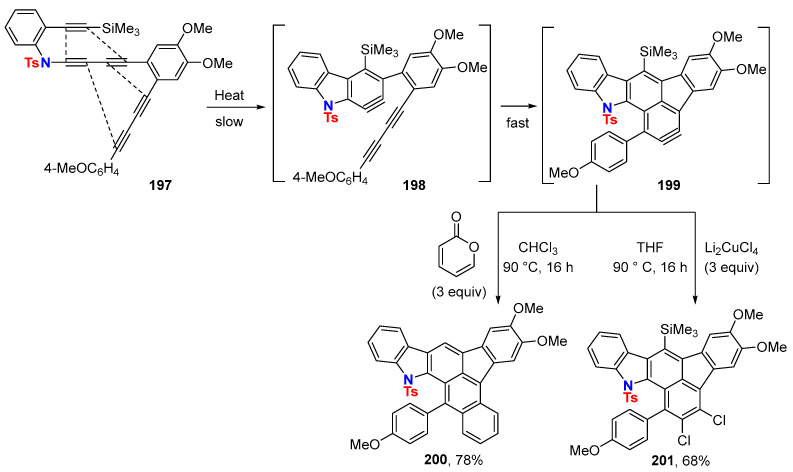
Domino HDDA reactions towards polyacenes.

Acenes with indolylnaphthalene or indolylanthracene units are accessible through iterative intramolecular HDDA reaction cascades starting from 1,3-butadiynamide **197** (Scheme 55) [87]. A first intramolecular HDDA cycloaddition gives aryne **198** consecutively undergoing a second intramolecular HDDA reaction to provide the new aryne **199** as the reactive intermediate. The latter is trapped either by [4+2] cycloaddition with α-pyrone to give indolylanthracene **200** or by dichlorination with dilithium tetrachlorocuprate, leading to indolylnaphthalene **201**, respectively, as the sole products.

**Scheme 56:**
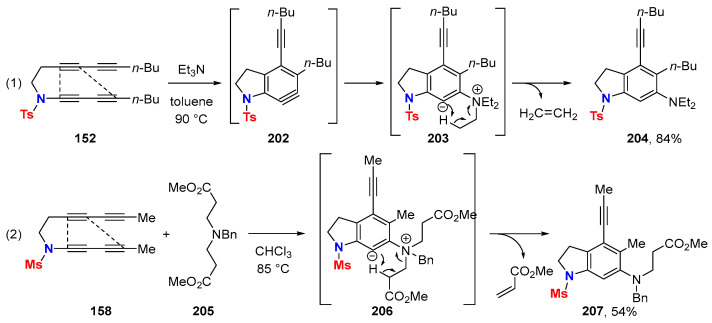
Tertiary amine addition onto HDDA-generated aryne.

*Aryne trapping* via *C-N bond formation*: In situ trapping of HDDA-generated aryne **202** with simple tertiary amines such as triethylamine leads to the zwitterionic species **203**, which delivers the same product **204** as being provided from **152** with diethylamine. Disproportionation of the zwitterion **203** by release of ethylene delivers the final product **204** (Scheme 56, (1)) [75]. This olefin elimination sequence was confirmed in the case of tertiary amines bearing activated *β*-protons such as *β*-aminoester **205** (Scheme 56, (2)) [88].

**Scheme 57:**
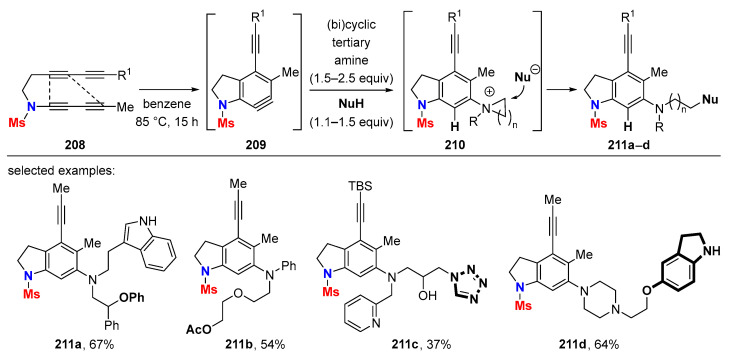
Three-component reactions of HDDA-generated benzynes with (bi)cyclic tertiary amines and protic nucleophiles.

However, in protic solvents, the preferential pathway does not involve intra- but intermolecular protonation of the zwitterionic species (**209** → **210**). Now, a quaternary ammonium center is formed following ring opening by a nucleophile in the case of cyclic tertiary amines (**210** → **211**) [88]. This reaction sequence also delivers poly-heterocyclic structures **211a**–**d** based on three-component reactions (TCR) with HDDA-generated arynes **209** (Scheme 57) [31]. The addition of the amine onto benzyne **209** must be faster than that of the protic nucleophile. In some cases, such as acetic acid, the expected TCR product **211b** (54%) was formed along with the direct benzyne–acetate addition product (27%). Thus, a two-step sequence based on the addition of triflic acid to generate ammonium triflates and their subsequent nucleophilic ring opening by nucleophilic displacement of the triflate in the second step broadens the scope of suitable nucleophiles.

**Scheme 58:**
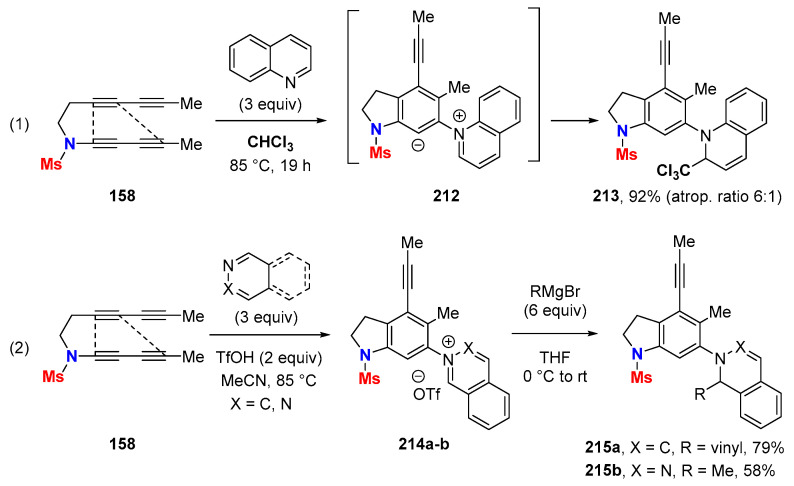
Reaction of HDDA-generated arynes with six-membered *N*-heteroaromatics.

The TCR strategy is applicable to six-membered *N*-heteroaromatics as the nitrogen nucleophile. For instance, the reaction of 1,3-butadiynamide **158** with quinoline in chloroform provides the addition product **213** via betaine **212** (Scheme 58, (1)) [89]. Catching the aryne generated from **158** with triflic acid and *N*-heteroaromatics like quinoline or quinazolines delivers isolable *N*-arylinium triflates **214a**–**b**, which undergo nucleophilic addition of Grignard reagents in a second reaction to give the heterocyclic products **215a**–**b** (Scheme 58, (2)).

**Scheme 59:**
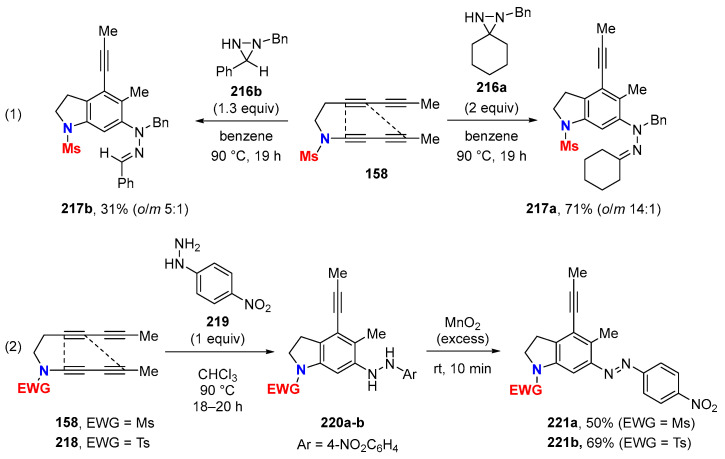
Trapping of HDDA-generated benzynes with (**1**) diaziridines and (**2**) arylhydrazines.

The reaction of HDDA-generated benzyne starting from 1,3-butadiynamide **158** and diaziridines **216a**–**b** delivers *N*-arylhydrazones **217a**–**b** (Scheme 59, (1)) [90]. The more nucleophilic but also more hindered *N*-benzyl nitrogen atom in **216a**–**b** adds to the aryne moiety. This reasons the formation of *meta*-isomer of **217a**–**b** along with the expected major *ortho*-isomer.

Arylhydrazines are ambident nucleophiles, but in the case of *para*-nitro-phenylhydrazine **219**, only the *β*-nitrogen atom (NH_2_) engages in the trapping of arynes thermally generated from tetraynes **158** (EWG = Ms) or **218** (EWG = Ts) (Scheme 59, (2)) [91]. The reaction is highly regioselective and subsequent oxidation of the resulting 1,2-diarylhydrazines **220a**–**b** with MnO_2_ delivers azoarene products **221a**–**b**.

**Scheme 60:**
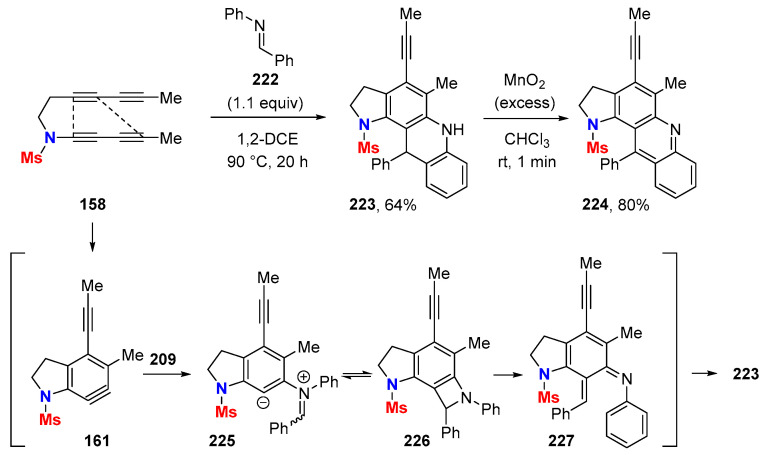
Trapping of HDDA-generated benzynes with a *C*,*N*-diarylimine.

The reaction of HDDA-generated arynes with *C*,*N*-diarylimine **222** proceeds efficiently, whereas this reaction is known to be poor yielding with *o*-benzynes generated by classical methods, i.e., by *ortho*-elimination of arene compounds (Scheme 60) [92]. Aromatization of the resulting dihydroacridine **223** by oxidation with MnO_2_ gives acridine **224**. The conversion of **158** to **223** probably involves a formal [2+2] cycloaddition of an imine to a highly reactive aryne species. Initial imine addition on benzyne **161** provides betaine **225**, which cyclizes to benzazetidine intermediate **226** to provide azo-quinomethide **227** after electrocyclic ring opening. Finally, a 6π electrocyclization followed by a proton shift gives dihydroacridine **223**.

**Scheme 61:**
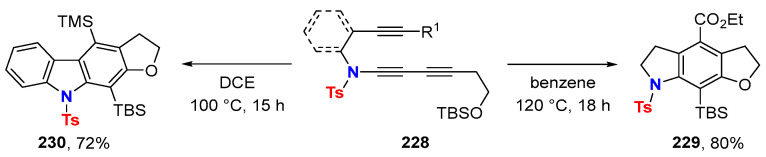
Intramolecular reaction of HDDA-generated benzynes from 1,3-butadiynamides tethered with a silyl ether.

*Aryne trapping* via *C-O bond formation*: Intramolecular HDDA reactions generated with 1,3-butadiynamide **228**, linked via two carbons to a silyl ether unit, result in fully substituted benzene derivatives via formal aryne insertion into the *O*-silicon bond. For example, furanyl-annulated indoline **229** and carbazole **230** are products from aryne reactions involving C-O bond formation (Scheme 61) [18,76].

**Scheme 62:**
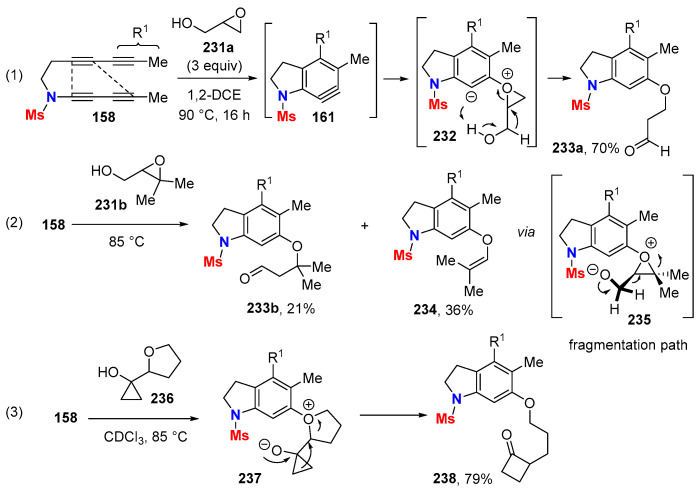
Diversity of HDDA–aryne reaction modes with glycidol derivatives via Pinacol-like rearrangements or oxirane fragmentation.

Intermolecular trapping of HDDA-generated benzynes from tetrayne **158** with hydroxy-containing cyclic ethers such as glycidol **231a** is also possible (Scheme 62, (1)) [93]. The reaction preferentially proceeds via the addition of the cyclic ether oxygen to the aryne. The resulting betaine **232** is protonated followed by ring opening of the cyclic oxonium ion delivering aryl ether **233a**. Addition of the alcohol hydroxy of **231a** to the aryne becomes a competitive pathway when a large excess of glycidol (100 equiv) is used. In the case of trisubstituted epoxide **231b**, another concurrent pathway based on C–C-bond cleavage and aldehyde liberation is observed leading to aryl enol derivative **234** (Scheme 62, (2)). The use of the cyclopropanol derivative **236** as the nucleophile readily delivered the cyclobutanone compound **238** via a cationic-driven cyclopropanol to the cyclobutanone ring-enlargement reaction (Scheme 62, (3)).

**Scheme 63:**
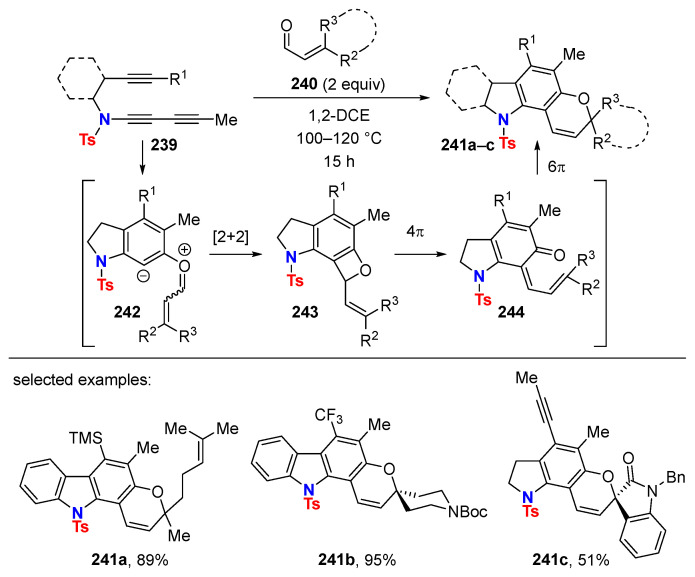
Catching HDDA-generated arynes with conjugated enals towards benzopyran structures.

HDDA reaction-generated arynes trapped by 3,3-disubstituted enals **240** offer a new entry to benzopyran motifs, in particular to the pyranocarbazole skeleton, as found in the products **241a**–**b** (Scheme 63) [76]. Betaine **242** is the primary addition product and undergoes a formal [2+2] cycloaddition to the benzoxetene **243**. Subsequent 4π-electrocyclic ring opening gives (*Z*)-dienone **244**, which finally undergoes 6π-electrocyclic ring closure to restore the aromaticity and gives the pyran moiety. The use of exocyclic conjugated enals delivered a variety of fused spirocyclic benzopyran structures like **241c** [94].

**Scheme 64:**
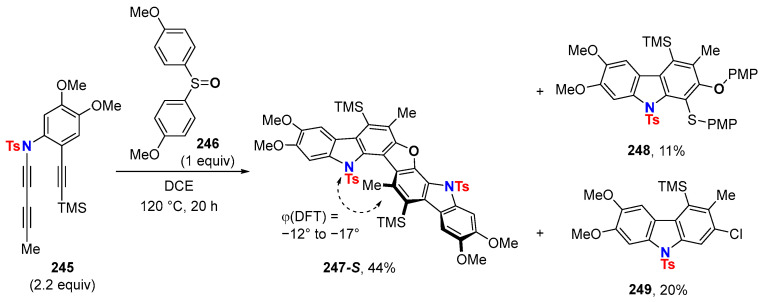
Synthesis of dimeric dibenzofuran helicenes.

With di(4-methoxy)phenyl sulfoxide (**246**) as an aryne-trapping agent, dimeric dibenzofuran *S*-shaped helicene **247** is obtained as major product along with carbazoles **248** and **249** starting from triyne **245** (Scheme 64) [95]. Remarkably, five new fused rings assemble within a single step under sole thermal activation.

**Scheme 65:**
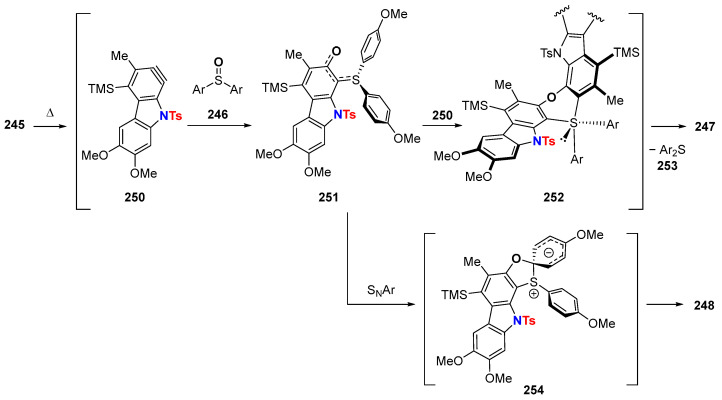
Mechanistic rationale for the formation of dibenzohelicene **247**.

Mechanistically, the reaction of HDDA-generated benzyne **250** with sulfoxide **246** first leads to adduct **251** (Scheme 65). The latter interacts with a second benzyne molecule **250** to give tetracarbo-ligated σ-sulfurane **252**. The hypervalent S(IV) species **252** is the key intermediate of this novel process. Reductive elimination of diarylsulfide **253** delivers the helicene product **247**. The 1:1 adduct carbazole **248** is the S_N_Ar reaction product of **251** via the Meisenheimer intermediate **254** (Scheme 65).

**Scheme 66:**
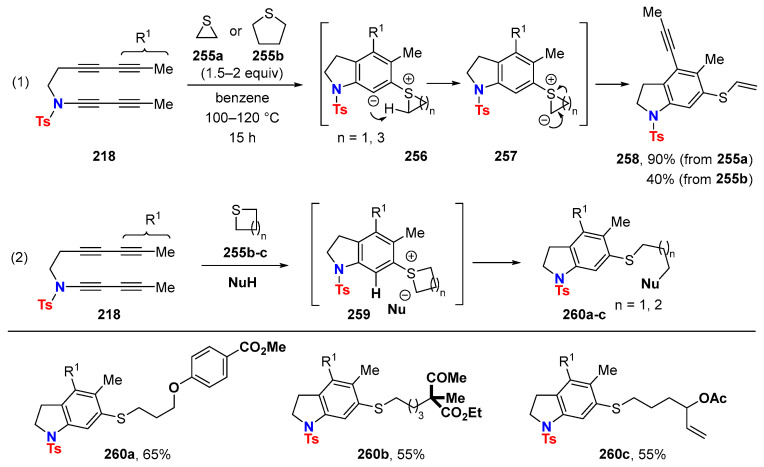
Reaction of HDDA-generated arynes with cyclic sulfides.

*Aryne trapping* via *C-S bond formation*: HDDA reactions of tetrayne **218** with its 1,3-butadiynamide moiety in the presence of either thiirane (**255a**) or tetrahydrothiophene **255b** both furnish vinyl sulfide **258** (Scheme 66, (1)) [96]. The initial formation of a betaine **256** (*o*-sulfonium/arylcarbanion) is followed by intramolecular proton transfer leading to a more stable *S*-aryl sulfur ylide **257**, which undergoes ring cleavage. Moreover, in the presence of a protic nucleophile, a three-component reaction takes place (Scheme 66, (2)). This becomes possible when the sulfide reacts faster with the aryne than the protic nucleophile. Intermolecular protonation of the resulting aryl carbanion leads to sulfonium **259**. Subsequent ring opening by nucleophile addition affords the final products **260a**–**c**. It is worthy of note that thiirane (**255a**) is not a suitable component for such a process because fragmentation to the vinyl sulfide is faster than nucleophile addition.

**Scheme 67:**
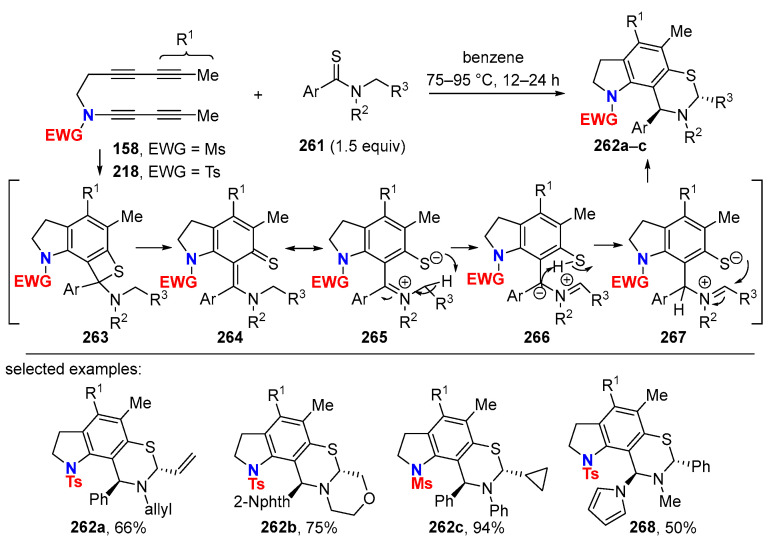
Reaction of HDDA-generated arynes with aromatic thioamides.

The HDDA reaction of tetraynes **158** (EWG = Ms) or **218** (EWG = Ts) with aryl thioamides **261** affords the dihydrobenzothiazine-derived molecules **262a**–**c** in a regio- and diastereoselective fashion (Scheme 67) [97]. Notably, these are the first examples of the use of thioamides as aryne-trapping agents. The proposed mechanism involves the formation of benzothietene **263** and its ring opening to give intermediate **264** or its resonance form *o*-thiolatoaryliminium **265**. Intramolecular 1,3-hydrogen atom migration delivers the iminium zwitterion **267**, whose cyclization leads to products **262a**–**c**. The analogous product **268** was also obtained using a thiourea as reaction partner.

**Scheme 68:**
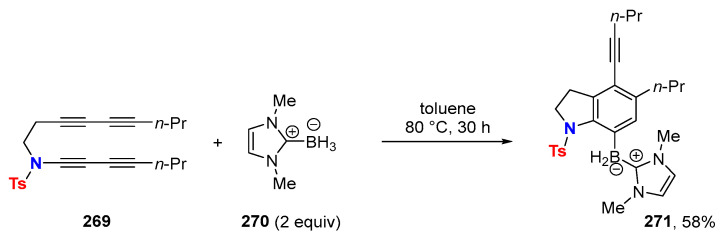
Trapping of HDDA-generated arynes with NHC-boranes.

*Aryne trapping* via *C-B bond formation*: The hydroboration of the aryne that was generated by an HDDA reaction with tetrayne **269**, with the *N*-heterocyclic carbene borane (NHC-borane) **270**, provides the highly functionalized arylborane compound **271** as a single isomer (Scheme 68) [98]. The feasibility of the reaction relies on the fact that NHC-boranes are deactivated and do not hydroborate the starting tetrayne unlike most other borane reagents.

### 6.2. Silver-Catalyzed HDDA Reactions

**Scheme 69:**
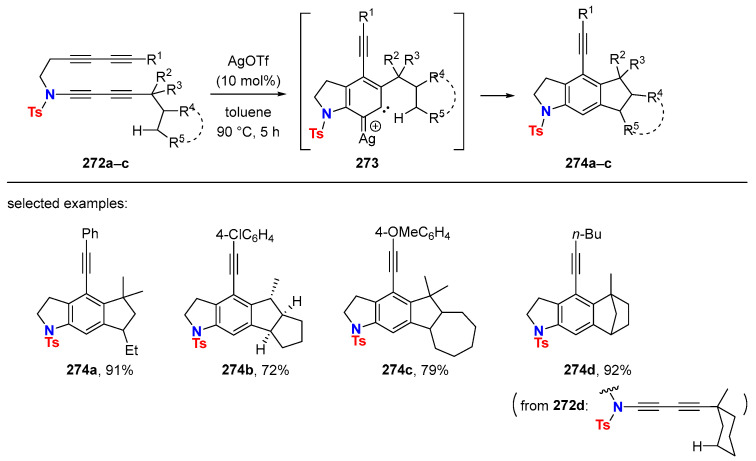
Reaction of HDDA-generated aryne via silver-catalyzed alkane C-H insertion.

*Ag(I)-bound aryne trapping* via *C-C bond formation*: Alkane C-H bonds can be activated by 1,3-butadiyne-HDDA-reaction generated arynes in the presence of silver(I) salts (Scheme 69) [99]. Importantly, under Ag(I)-free reaction conditions, the alkane C-H insertion fails to appear. According to mechanistic studies, the C(sp^3^)-H bond-breaking and C(sp^2^)-H bond-forming processes are concerted and induced via the silver-carbenoid intermediate **273**.

**Scheme 70:**
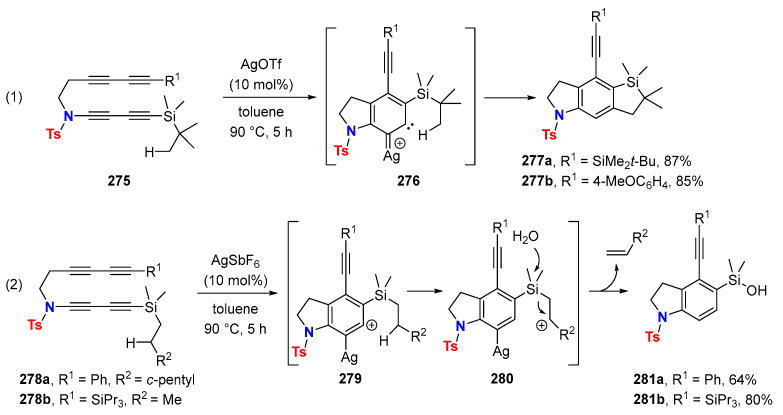
Intramolecular trapping of HDDA-generated aryne from silyl-substituted substrates.

The fact that Ag(I) salts drive the character of the aryne reactivity from an alkyne towards a carbene character was nicely shown with the silyl-substituted substrates **275** bearing a primary C-H bond (Scheme 70, (1)) [100]. In the presence of a secondary or tertiary C-H bond on the *β*-carbon of the silyl group, hydride transfer occurs instead of C-H insertion (Scheme 70, (2)). This result is plausible considering a favorable hyperconjugation of the *β*-silicon stabilized carbocation **280**, whose reaction with water delivers the formal aryne hydrogenation products **281a**–**b**.

**Scheme 71:**
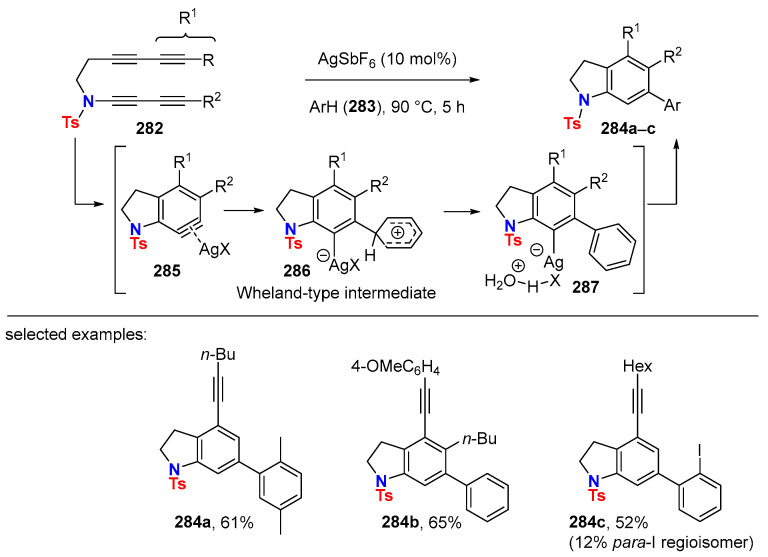
Intermolecular hydroarylation of silver-complexed HDDA-generated arynes.

Silver-catalyzed reactions of non-activated benzenes **283** with 1,3-butadiynamide-**282**–HDDA reaction-generated arynes lead to the hydroarylation products **284a**–**c** (Scheme 71) [101]. Diels–Alder reaction products of arynes with arenes, as usually found with “free arynes”, stay out and are not formed. The chemical outcome of this transformation is rationalized by an electrophilic aromatic substitution through the formation of a Wheland-type intermediate **286** (arenium ion) and subsequent water-catalyzed proton transfer.

**Scheme 72:**
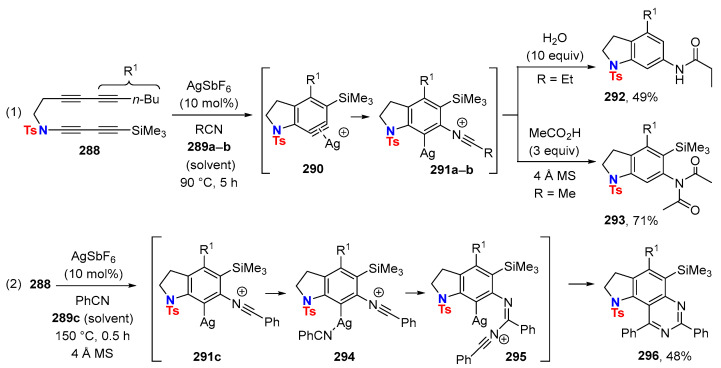
Reaction of silver-complexed HDDA-generated arynes with nitriles.

*Ag(I)-bound aryne trapping* via *C-N bond formation*: Nitriles are too weak nucleophiles to react with arynes. However, in the presence of a silver catalyst, the trapping of HDDA-generated aryne is taking place with nitriles readily via the formation of nitrilium ion intermediates **291a**–**b**. The latter can react with water or with acetic acid under anhydrous conditions to give indolinyl amide **292** or imide **293**, respectively, starting from tetrayne **288** (Scheme 72, (1)) [102].

Under anhydrous conditions and in the absence of carboxylic acids, tetrayne **288** converts into quinazoline **296** by incorporation of two benzonitrile molecules (**289c**) (Scheme 72, (2)) [103]. Now, the nitrilium ion **291c** interacts with a second nitrile molecule to give the resonance-stabilized complex **294**. Transformation of **294** into *bis*-nitrile adduct **295** and the subsequent ring closure finally delivers quinazoline **296**.

**Scheme 73:**
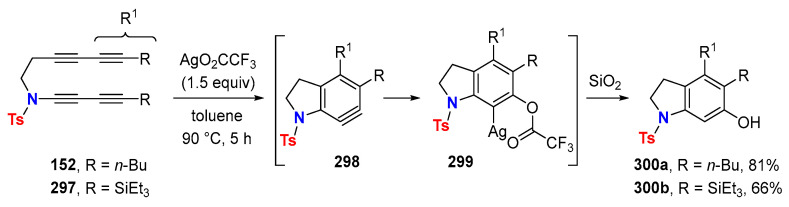
Synthesis of phenolic compounds from HDDA-generated arynes.

*Ag(I)-bound aryne trapping* via *C-O bond formation*: Water, unlike alcohols or carboxylic acids, is a less suitable aryne-trapping agent to deliver subsequent phenols. This is probably because of the immiscibility of water in organic solvents where transient arynes are generated. Silver trifluoroacetate (AgO_2_CCF_3_) turned out to be a suitable water surrogate to obtain phenol-related compounds [75,104]. Indeed, the reaction of 1,3-butadiynes **152** or **297** with AgO_2_CCF_3_ leads via transient HDDA reaction-generated arynes **298** to trifluoroacetoxy organosilver arenes **299**, whose hydrolysis on silica gel furnishes the phenolic compounds **300a**–**b** (Scheme 73).

**Scheme 74:**
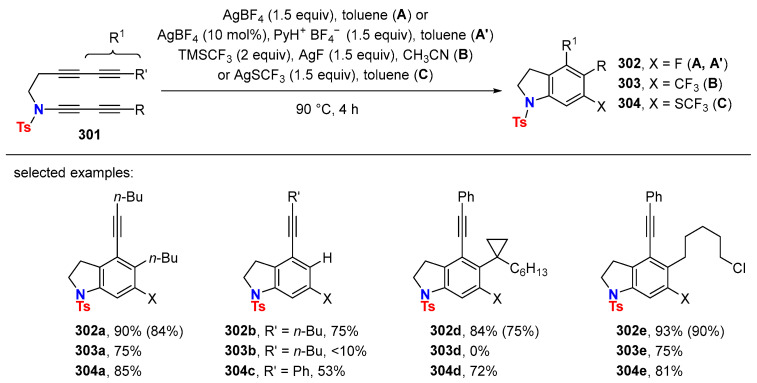
Silver-mediated fluorination, trifluoromethylation and trifluoromethylthiolation of HDDA-generated arynes.

Ag(I)-bound aryne trapping with fluorine-containing reagents: Fluorination, trifluoromethylation and trifluoromethylthiolation of HDDA-generated arynes from 1,3-butadiynes **301** were successfully achieved by using AgBF_4_, AgCF_3_ (in situ generated from AgF and TMSCF_3_) and AgSCF_3_ in stoichiometric quantities, respectively (Scheme 74) [105]. The trifluoromethylation reaction is not proceeding with terminal (R = H) or tertiary alkyl-substituted 1,3-butadiynamides as starting materials (see **303b**, **303d**). A catalytic protocol for aryne fluorination appeared. It relies on the use of AgBF_4_ (10 mol%) as the catalyst and the BF_4_•pyridinium salt (1.5 equiv) as the fluoride source. Importantly, fluorination fails in the absence of silver salts.

### 6.3. HDDA Reactions Catalyzed by Other Metals Than Silver

**Scheme 75:**
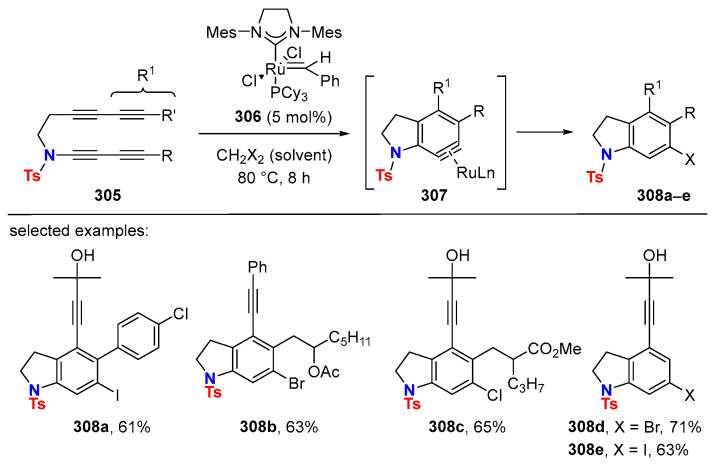
Ruthenium-catalyzed hydrohalogenation of HDDA-generated arynes.

The hydrohalogenative aromatization of ynamide-derived tetraynes **305** readily proceeds in halogenated hydrocarbons CH_2_X_2_ (X = Cl, Br, I) as the solvent in the presence of Grubbs-type ruthenium alkylidene complex **306** and delivers novel halogenated indoline derivatives **308a**–**e** (Scheme 75) [106]. The proposed mechanism involves the activation of CH_2 × 2_ by the ruthenium catalyst for the HX transfer to ruthenium-complexed aryne intermediate **307**.

**Scheme 76:**
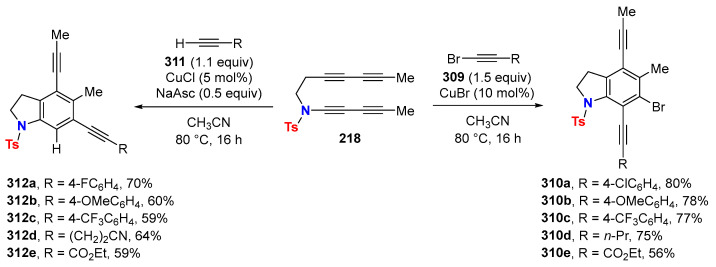
Copper-catalyzed bromo- and hydroalkynylation of HDDA-generated arynes.

The addition of 1-bromoalkynes **309** or terminal alkynes **311** to 1,3-butadiyne-**218**-HDDA-reaction generated aryne proceeds efficiently under copper catalysis (Scheme 76) [107]. Interestingly, the insertion of the alkynyl moiety into the aryne carbon–carbon triple bond to give either indolines **310a**–**e** or **312a**–**e**, respectively, takes place at complementary positions.

**Scheme 77:**
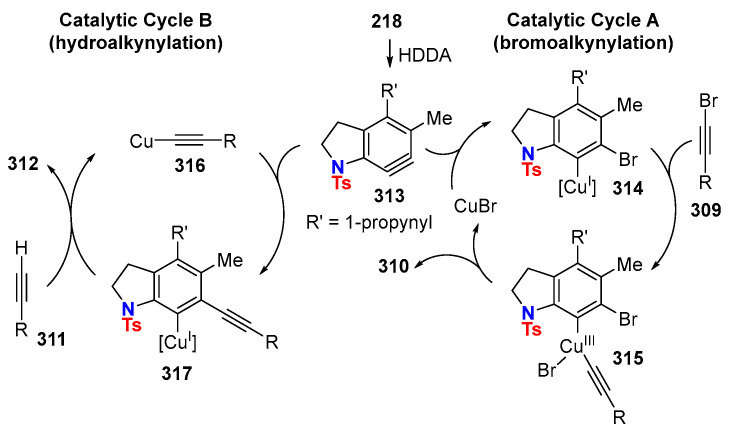
Proposed mechanism of copper-catalyzed bromoalkynylation and hydroalkynylation of HDDA-generated arynes.

Two different catalytic cycles **A** and **B** are proposed to rationalize the outcome of these reactions (Scheme 77). In the case of the bromoalkynylation, CuBr adds to in situ generated aryne **313** (from 1,3-diynamide **218**) to give aryl copper (I) species **314**. Oxidative addition of this Cu(I) species to 1-bromoalkyne **309** gives the aryl copper(III) intermediate **315**. Subsequent reductive elimination produces the 4,7-diethynyldihydroindole **310** and regenerates the copper(I) catalyst. In the case of the catalytic hydroalkynylation reaction (catalytic cycle B), aryne **313** undergoes carbocupration with in situ generated copper acetylide **316** from terminal alkyne **311**, providing aryl copper species **317**. Proton exchange with terminal alkyne **311** delivers compound **312** and closes the catalytic cycle.

**Scheme 78:**
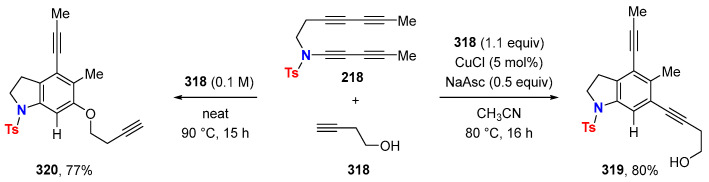
Different outcomes of the reaction of HDDA-generated aryne with homopropargyl alcohol with and without copper catalyst.

The catalytic reaction of tetrayne **218** with homopropargyl alcohol (**318**) selectively gives the hydroalkynylation product **319**, whereas compound **320** resulting from the addition of the alcohol moiety on the transient aryne **313** is the major compound in the absence of copper catalyst (Scheme 78).

## 7. Conclusions and Perspectives

The present review covers the synthesis, molecular properties and use of 1,3-butadiynamides in heterocyclic chemistry. Based on this comprehensive summary and its compiled reactions, which not only focused on applications in organic synthesis, but also on mechanistic aspects, it becomes obvious that 1,3-butadiynamides—the ethynylogous variant of ynamides—are more than just simple alkynes: they should be considered as a new class of compounds showing its own specific reactivity.

Their potential as building blocks for the construction of complex molecular scaffolds has emerged only recently, although the synthesis of symmetrical and unsymmetrical 1,3-butadiynamides was described more than 15 years ago. Terminal ynamides are useful precursors of 1,3-butadiynamides for their use in the Glaser–Hay coupling reaction or the Cadiot–Chodkiewicz cross-coupling reaction with 1-bromoalkynes. The latter emerged to a broadly used and indispensable synthetic method to access various highly functionalized non-symmetrical 1,3-butadiynamides. Alternative protocols based on the direct *N*-buta-1,3-diynylation of amides appeared just recently and will find further attention.

The extended conjugation and polarized character of 1,3-butadiynamides facilitate their use in addition and cycloaddition reactions with predictable regioselectivity. Many of them involve gold catalysis along with the successful use of other metal salts (Pd, Ag, Cu/Zn). Moreover, 1,3-butadiynamides are easily derivatized by introducing judicious functional groups tethered to the *N*-atom and/or to the C-terminal of the 1,3-diynyl moiety. Such diversely functionalized 1,3-butadiynamides serve as highly useful molecular scaffolds in the development of new reaction cascades.

Intensive studies have been dedicated to thermal or metal-catalyzed reactions with 1,3-butadiynamides tethered to a monoalkyne or 1,3-butadiyne moiety. These tetra- or triynes having a 1,3-butadiynamide unit are privileged substrates for the HDDA reaction because they lead to the in situ formation of a single aryne isomer, and its intermolecular trapping takes place regioselectively. Reagent-free, highly chemo- and regioselective aryne formation with regioselective follow-up reactions by inter- or intramolecular aryne trapping not only enhanced the chemistry of 1,3-butadiynamides, but also boosted the understanding of different reaction channels available for in situ generated aryne species. HDDA reactions with 1,3-butadiynamide units can be performed solely thermally or in the presence of sub-stoichiometric amounts of Ag(I)-salts. The latter often changes the mode of reactivity towards a more carbenoid character of the aryne intermediate and opens new alternative reaction channels.

Most described 1,3-butadiynamides are derived from sulfonamides. It is conceivable that the synthesis of carbamate- or oxazolidinone-related 1,3-butadiynamides will be further explored and that new reaction sequences will be discovered, as the strength of the nitrogen bound EWG group will alter the 1,3-butadiynamide reactivity.

The more classical activation of 1,3-butadiynamides relies on the in situ generation of a keteniminium species to accelerate follow-up transformations. Recently, a new reaction mode—the in situ liberation of [4]cumulenenimines from 1,3-butadiynamides—was revealed. This finding will probably initiate the development of novel reaction cascades and broaden the array of available structurally complex molecules.

Finally, the examples of optically active 1,3-butadiynamides are scarce, and the possibility of chirality transfer during reaction cascades has not been investigated yet. Asymmetric or enantioselective synthesis relying on the transformation of 1,3-butadiynamides will certainly be a future topic to gain access to the chiral world of heterocycles. Surely, the chemistry of 1,3-butadiynamides is still at its infancy and more new reactions and more sophisticated applications, especially in the field of heterocyclic chemistry, will appear in near future.

## Figures and Tables

**Table 1:** Electrooptical absorption measurement (EOAM) of selected “push–pull” ynamides end-capped with a nitrogen donor and a 4-nitro-phenyl acceptor unit [25].

Ynamide	*μ*_g_	Δ^a^*μ*	*μ*_a_^FC^
	[10^−30^ Cm] in 1,4-dioxane		
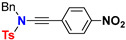	9.7	74.6	84.3
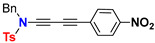	9.5	92.3	101.8
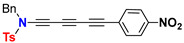	10.9	30.8	41.7
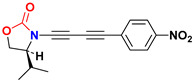	11.8	69.7	81.5

## Data Availability

Not applicable.

## References

[1] Witulski B., Stengel T. (1998). *N*-Functionalized 1-Alkynylamides: New Building Blocks for Transition Metal Mediated Inter- and Intramolecular [2+2+1] Cycloadditions. Angew. Chem. Int. Ed..

[2] Iftikhar R., Mazhar A., Iqbal M.S., Khan F.Z., Askary S.H., Sibtain H. (2023). Ring Forming Transformation of Ynamides via Cycloaddition. RSC. Adv..

[3] Hu Y.-C., Zhao Y., Wan B., Chen Q.-A. (2021). Reactivity of Ynamides in Catalytic Intermolecular Annulations. Chem. Soc. Rev..

[4] Shandilya S., Gogoi M.P., Dutta S., Sahoo A.K. (2021). Gold-Catalyzed Transformation of Ynamides. Chem. Rec..

[5] Campeau D., Rayo D.F.L., Mansour A., Muratov K., Gagosz F. (2021). Gold-Catalyzed Reactions of Specially Activated Alkynes, Allenes and Alkenes. Chem. Rev..

[6] Chen Y.-B., Qian P.-C., Ye L.-W. (2020). Brønsted Acid-Mediated Reactions of Ynamides. Chem. Soc. Rev..

[7] Lynch C.C., Sripada A., Wolf C. (2020). Asymmetric Synthesis with Ynamides: Unique Reaction Control, Chemical Diversity and Applications. Chem. Soc. Rev..

[8] Tan T.-D., Wang Z.-S., Qian P.-C., Ye L.-W. (2021). Radical Reactions of Ynamides. Small Methods.

[9] Mahe C., Cariou K. (2020). Ynamides in Free Radical Reactions. Adv. Synth. Catal..

[10] Hong F.-L., Ye L.-W. (2020). Transition Metal-Catalyzed Tandem Reactions of Ynamides for Divergent *N*-Heterocycle Synthesis. Acc. Chem. Res..

[11] Zhou B., Tan T.-D., Zhu X.-Q., Shang M., Ye L.-W. (2019). Reversal of Regioselectivity in Ynamide Chemistry. ACS Catal..

[12] Prabagar B., Ghosh N., Sahoo A.K. (2017). Cyclization and Cycloisomerization of π-Tethered Ynamides: An Expedient Synthetic Method to Construct Carbo- and Heterocycles. Synlett.

[13] Cook A.M., Wolf C. (2015). Terminal Ynamides: Synthesis, Coupling Reactions, and Additions to Common Electrophiles. Tetrahedron Lett..

[14] Wang X.-N., Yeom H.-S., Fang L.-C., He S., Ma Z.-X., Kedrowski B.L., Hsung R.P. (2014). Ynamides in Ring Forming Transformations. Acc. Chem. Res..

[15] Evano G., Coste A., Jouvin K. (2010). Ynamides: Versatile Tools in Organic Synthesis. Angew. Chem. Int. Ed..

[16] DeKorver K.A., Li H., Lohse A.G., Hayashi R., Lu Z., Zhang Y., Hsung R.P. (2010). Ynamides: A Modern Functional Group for the New Millennium. Chem. Rev..

[17] Fluegel L.L., Hoye T.R. (2021). Hexadehydro-Diels–Alder Reaction: Benzyne Generation via Cycloisomerization of Tethered Triynes. Chem. Rev..

[18] Hoye T.R., Baire B., Niu D., Willoughby P.H., Woods B.P. (2012). The hexadehydro-Diels–Alder reaction. Nature.

[19] Holden (née Hall) C., Greaney M.F. (2014). The Hexadehydro-Diels–Alder Reaction: A New Chapter in Aryne Chemistry. Angew. Chem. Int. Ed..

[20] Diamond O.J., Marder T.B. (2017). Methodology and Applications of the Hexadehydro-Diels-Alder (HDDA) Reaction. Org. Chem. Front..

[21] Yu H., Xu F. (2023). Advances in the Synthesis of Nitrogen-Containing Heterocyclic Compounds by in situ Benzyne Cycloaddition. RSC Adv..

[22] Rodriguez D., Castedo L., Saa C. (2004). Homocoupling of 1-Alkynyl Tosylamides. Synlett.

[23] Doan T.-H., Talbi I., Lohier J.-F., Touil S., Alayrac C., Witulski B. (2016). Synthesis, Crystal Structure, Optical, Electrochemical and Thermal Properties of the Ynamide: Bis-(*N*-4-methylbenzenesulfonyl, *N*-n-butyl)-1,3-butadiyne-1,4-diamide. J. Mol. Struct..

[24] Takai R., Shimbo D., Tada N., Itoh A. (2021). Ligand-Enabled Copper-Catalyzed *N*-Alkynylation of Sulfonamide with Alkynyl Benziodoxolone: Synthesis of Amino Acid-Derived Ynamide. J. Org. Chem..

[25] Witulski B., Schweikert T., Schollmeyer D., Nemkovich N.A. (2010). Synthesis and Molecular Properties of Donor-π-Spacer-Acceptor Ynamides with up to Four Conjugated Alkyne Units. Chem. Commun..

[26] Talbi I., Alayrac C., Lohier J.-F., Touil S., Witulski B. (2016). Application of Ynamides in the Synthesis of 2-(Tosylamido)- and 2,5-Bis(tosylamido)thiophenes. Org. Lett..

[27] Wang Y.-P., Danheiser R.L. (2011). Synthesis of 2-Iodoynamides and Regioselective [2+2] Cycloadditions with Ketene. Tetrahedron Lett..

[28] Tracey M.R., Zhang Y., Frederick M.O., Mulder J.A., Hsung R.P. (2004). Sonogashira Cross-Couplings of Ynamides. Syntheses of Urethane- and Sulfonamide-Terminated Conjugated Phenylacetylenic Systems. Org. Lett..

[29] Dunetz J.R., Danheiser R.L. (2003). Copper-Mediated *N*-Alkynylation of Carbamates, Ureas, and Sulfonamides. A General Method for the Synthesis of Ynamides. Org. Lett..

[30] Kohnen A.L., Dunetz J.R., Danheiser R.L. (2007). Synthesis of Ynamides by *N*-Alkynylation of Amine Derivatives. Preparation of *N*-Allyl-*N*-(Methoxycarbonyl)-1,3-Decadiynylamine. Org. Synth..

[31] Ross S.P., Hoye T.R. (2018). Multiheterocyclic Motifs via Three-Component Reactions of Benzynes, Cyclic Amines, and Protic Nucleophiles. Org. Lett..

[32] Zhang Y., Hsung R.P., Tracey M.R., Kurtz K.C.M., Vera E.L. (2004). Copper Sulfate-Pentahydrate-1,10-Phenanthroline Catalyzed Amidations of Alkynyl Bromides. Synthesis of Heteroaromatic Amine Substituted Ynamides. Org. Lett..

[33] Wang T., Niu D., Hoye T.R. (2016). The Hexadehydro-Diels–Alder Cycloisomerization Reaction Proceeds by a Stepwise Mechanism. J. Am. Chem. Soc..

[34] Mansfield S.J., Smith R.C., Yong J.R.J., Garry O.L., Anderson E.A. (2019). A General Copper-Catalyzed Synthesis of Ynamides from 1,2-Dichloroenamides. Org. Lett..

[35] Kawakarni R., Usui S., Tada N., Itoh A. (2023). Late-Stage Diversification Strategy for Synthesizing Ynamides through Copper-Catalyzed Diynylation and Azide-Alkyne Cycloaddition. Chem. Commun..

[36] Gohrai S., Lee D. (2021). Selectivity for Alkynyl or Allenyl Imidamides and Imidates in Copper-Catalyzed Reactions of Terminal 1,3-Diynes and Azides. Org. Lett..

[37] Garner M.H., Hoffmann R., Rettrup S., Solomon G.C. (2018). Coarctate and Möbius: The Helical Orbitals of Allene and Other Cumulenes. ACS Cent. Sci..

[38] Garner M.H., Jensen A., Hyllested L.O.H., Solomon G.C. (2019). Helical Orbitals and Circular Currents in Linear Carbon Wires. Chem. Sci..

[39] Garner M.H., Corminboeuf C. (2021). Helical Electronic Transitions of Spiroconjugated Molecules. Chem. Commun..

[40] Ohashi S., Inagaki S. (2001). Orbital Phase Control of Conformations of Alkyne Derivatives. Tetrahedron.

[41] Hendon C.H., Tiana D., Murray A.T., Carbery D.R., Walsh A. (2013). Helical Frontier Orbitals of Conjugated Linear Molecules. Chem. Sci..

[42] Balakrishnan A.R., Suresh R., Vijayakumar S. (2022). Amine Terminated Polyynes as Candidates for Molecular Wire Applications: A DFT Study. Phys. E Low-Dimens. Syst. Nanostruct..

[43] Bro-Jorgensen W., Garner M.H., Solomon G.C. (2021). Quantification of the Helicality of Helical Molecular Orbitals. J. Phys. Chem. A.

[44] Petrov A.R., Daniliuc C.G., Jones P.G., Tamm M. (2010). A Novel Synthetic Approach to Diaminoacetylenes: Structural Characterization and Reactivity of Aromatic an Aliphatic Ynediamines. Chem. Eur. J..

[45] Tokutome Y., Okuno T. (2013). Preparations, Crystal Polymorphs and DFT Calculations of N^1^,N^1^,N^4^,N^4^-Tetraphenylbuta-1,3-diyne-1,4-diamine. J. Mol. Struct..

[46] Mayerle J.J., Flandera M.A. (1978). Bis(1-carbazolyl)butadiyne. Acta Cryst..

[47] Matsuda H., Nakanishi H., Hosomi T., Kato M. (1988). Synthesis and Solid-State Polymerization of a New Diacetylene: 1-(*N*-Carbazolyl)penta-1,3-diyn-5-ol. Macromolecules.

[48] Tabata H., Kuwamoto K., Okuno T. (2016). Conformational Polymorphs and Solid-State Polymerization of 9-(1,3-Butadiynyl)carbazole Derivatives. J. Mol. Struct..

[49] Ide M., Ohashi K., Mihara S., Iwasaha T. (2014). Regio- and Stereoselective hydrohalogenation of Ynamide Components in 1,3-Butadiynes with in situ Generated HX. Tetrahedron Lett..

[50] De la Vega-Hernandez K., Romain E., Coffinet A., Bijouard K., Gontard G., Chemla F., Ferreira F., Jackowski O., Perez-Luna A. (2018). Radical Germylzincation of α-Heteroatom-Substituted Alkynes. J. Am. Chem. Soc..

[51] Kramer S., Madsen J.L.H., Rottländer M., Skrydstrup T. (2010). Access to 2,5-Diamidopyrroles and 2,5-Diamidofurans by Au(I)-Catalyzed Double Hydroamination or Hydration of 1,3-Diynes. Org. Lett..

[52] Choudhary S., Gayyur, Ghosh N. (2022). Cu(II)-Catalyzed [4+1] and [4+3] Annulation Reactions: A Modular Approach to *N*-Aryl/Alkyl Substituted 2,5-Diamidopyrroles and Diazepines. Org. Biomol. Chem..

[53] Dunetz J.R., Danheiser R.L. (2005). Synthesis of Highly Substituted Indolines and Indoles via Intramolecular [4+2] Cycloaddition of Ynamides and Conjugated Enynes. J. Am. Chem. Soc..

[54] Liao Y., Lu Q., Chen G., Yu Y., Li C., Huang X. (2017). Rhodium-Catalyzed Azide-Alkyne Cycloaddition of Internal Ynamides: Regioselective Assembly of 5-Amino-Triazoles under Mild Conditions. ACS Catal..

[55] Davies P.W., Cremonesi A., Dumitrescu L. (2011). Intermolecular and Selective Synthesis of 2,4,5-Trisubstituted Oxazoles by a Gold-Catalyzed Formal [3+2] Cycloaddition. Angew. Chem. Int. Ed..

[56] Skaria M., Hsu Y.-C., Jiang Y.-T., Lu M.-Y., Kuo T.-C., Cheng M.-J., Liu R.-S. (2020). Gold-Catalyzed Oxidations of 1,3-Diynamides with *C*(1) versus *C*(3) Regioselectivity: Catalyst-Dependent Oxidative Cyclizations in the C(3) Oxidation. Org. Lett..

[57] Lee D., Kim M. (2007). Advances in the Metallotropic [1,3]-Shift of Alkynyl Carbenoids. Org. Biomol. Chem..

[58] Yanai H., Kawazoe T., Ishii N., Witulski B., Matsumoto T. (2021). Regioselective Synthesis of 4-Aryl-1,3-dihydroxy-2-naphthoates through 1,2-Aryl-Migrative Ring Rearrangement Reaction and their Photoluminescence Properties. Chem. Eur. J..

[59] Kumar M., Kaliya K., Maurya S.K. (2023). Recent Progress in the Homogeneous Gold-Catalysed Cycloisomerisation Reactions. Org. Biomol. Chem..

[60] Liu J., Chakraborty P., Zhang H., Zhong L., Wang Z.-X., Huang X. (2019). Gold-Catalyzed Atom-Economic Synthesis of Sulfone-Containing Pyrrolo [2,1-a]isoquinolines from Diynamides: Evidence for Consecutive Sulfonyl migration. ACS Catal..

[61] Choudhary S., Gayyur, Ghosh N. (2023). Gold(I)-Catalyzed and PTSA-Promoted Cycloisomerization of Ynamides to Access Pyrrole Substituted α,β-Unsaturated Ketones. Eur. J. Org. Chem..

[62] Wang H.F., Guo L.N., Fan Z.B., Tang T.H., Zi W. (2021). Gold-Catalyzed Formal Hexadehydro-Diels–Alder/Carboalkoxylation Reaction Cascades. Org. Lett..

[63] Liu N., Sun H., Wang J., Zhang Z., Wang T. (2021). Ag(I)-Catalyzed Synthesis of 2-Aminoquinolines from 1-Aminobutadiynes and Anilines. Adv. Synth. Catal..

[64] Wang J., Du J., Wang T. (2023). Synthesis of 2-Aminopyrrolo[1,2-b]pyridazines via Gold(I)-Catalyzed Chemoselective Hydroamination/Hydroarylation Cascade of 1,3-Diynamides with 1-Aminopyrroles. Adv. Synth. Catal..

[65] Gayyur, Choudhary S., Kant R., Ghosh N. (2022). Synergetic Copper/Zinc Catalysis: Synthesis of Aryl/Heteroaryl-Fused 1*H*-Pyrrolo[3,2-*c*]pyridines. Chem. Commun..

[66] Pandit Y.B., Jiang Y.-T., Jian J.-J., Chen T.-C., Kuo T.-C., Cheng M.-J., Liu R.-S. (2021). Gold-Catalyzed [5+2]-Annulations of 1,3-Diyn-1-amides with Anthranils Bearing no C(6)-Substituents. Org. Chem. Front..

[67] Tian X., Song L., Farshadfar K., Rudolph M., Rominger F., Oeser T., Ariafard A., Hashmi A.S.K. (2020). Acyl Migration *versus* Epoxidation in Gold Catalysis: Facile, Switchable, and Atom-Economic Synthesis of Acylindoles and Quinoline Derivatives. Angew. Chem. Int. Ed..

[68] Liu J., Zhu L., Wan W., Huang X. (2020). Gold-Catalyzed Cascade Cyclization of 1,3-Diynamides: Polycyclic N-Heterocycle Synthesis via Construction of a Furopyridinyl Core. Org. Lett..

[69] Xia J., Liu J., Yu Y., Zhang J., Huang X. (2022). Divergent Access to Polycyclic *N*-Heterocyclic Compounds through Büchner-Type Dearomatisation Enabled Cycloisomerization of Diynamides under Gold Catalysis. Org. Lett..

[70] Lenko I., Mamontov A., Alayrac C., Legay R., Witulski B. (2021). Media-Driven Pd-Catalyzed Reaction Cascades with 1,3-Diynamides Leading Selectively to Either Indoles or Quinolines. Angew. Chem. Int. Ed..

[71] Witulski B., Alayrac C., Tevzadze-Saeftel L. (2003). Palladium-Catalyzed Synthesis of 2-Aminoindoles by a Heteroannulation Reaction. Angew. Chem. Int. Ed..

[72] Bradley A.Z., Johnson R.P. (1997). Thermolysis of 1,3,8-Nonatriyne: Evidence for Intramolecular [2+4] Cycloaromatization to a Benzyne Intermediate. J. Am. Chem. Soc..

[73] Miyawaki K., Suzuki R., Kawano T., Ueda I. (1997). Cycloaromatization of a Non-Conjugated Polyenyne System: Synthesis of 5*H*-Benzo[*d*]fluoreno[3,2-*b*]pyrans via Diradicals Generated from 1-[2-{4-(2-Alkoxymethylphenyl)butan-1,3-diynyl}]phenylpentan-2,4-diyn-1-ols and Trapping Evidence for the 1,2-Didehydrobenzene Diradical. Tetrahedron Lett..

[74] Liang Y., Hong X., Yu P., Houk K.N. (2014). Why Alkynyl Substituents Dramatically Accelerate Hexadehydro-Diels–Alder (HDDA) Reactions: Stepwise Mechanisms of HDDA Cycloadditions. Org. Lett..

[75] Karmakar R., Yun S.Y., Wang K.-P., Lee D. (2014). Regioselectivity in the Nucleophile Trapping of Arynes: The Electronic and Steric Effects of Nucleophiles and Substituents. Org. Lett..

[76] Wang T., Hoye T.R. (2016). Hexadehydro-Diels–Alder (HDDA)-Enabled Carbazolyne Chemistry: Single Step, de Novo Construction of the Pyranocarbazole Core of Alkaloids of the *Murraya koenigii* (Curry Tree) Family. J. Am. Chem. Soc..

[77] Ross S.P., Hoye T.R. (2017). Reactions of Hexadehydro-Diels–Alder Benzynes with Structurally Complex Multifunctional Natural Products. Nat. Chem..

[78] Chen M., He C.Q., Houk K.N. (2019). Mechanism and Regioselectivity of an Unsymmetrical Hexadehydro-Diels–Alder (HDDA) Reaction. J. Org. Chem..

[79] Cheong P.H.-Y., Paton R.S., Bronner S.M., Im G.-Y.J., Garg N.K., Houk K.N. (2010). Indolyne and Aryne Distortions and Nucleophilic Regioselectivities. J. Am. Chem. Soc..

[80] Karmakar R., Lee D. (2016). Reactions of Arynes Promoted by Silver Ions. Chem. Soc. Rev..

[81] Zhang J., Niu D., Brinker V.A., Hoye T.R. (2016). The Phenol–Ene Reaction: Biaryl Synthesis via Trapping Reactions between HDDA-Generated Benzynes and Phenolics. Org. Lett..

[82] Gupta S., Xie P., Xia Y., Lee D. (2017). Reactivity and Selectivity in the Intermolecular Alder–Ene Reactions of Arynes with Functionalized Alkenes. Org. Lett..

[83] Karmakar R., Mamidipalli P., Yun S.Y., Lee D. (2013). Alder-Ene Reactions of Arynes. Org. Lett..

[84] Karmakar R., Le A., Xie P., Xia Y., Lee D. (2018). Reactivity of Arynes for Arene Dearomatization. Org. Lett..

[85] Gupta S., Lin Y., Xia Y., Wink D.J., Lee D. (2019). Alder-Ene Reactions Driven by High Steric Strain and Bond Angle Distortion to Form Benzocyclobutenes. Chem. Sci..

[86] Le A., Lee D. (2021). Selectivity between an Alder-ene Reaction and a [2+2] Cycloaddition in the Intramolecular Reactions of Allene-Tethered Arynes. Org. Chem. Front..

[87] Xiao X., Hoye T.R. (2018). The Domino Hexadehydro-Diels–Alder Reaction Transforms Polyynes to Benzynes to Naphthynes to Anthracynes to Tetracynes (and Beyond?). Nat. Chem..

[88] Ross S.P., Baire B., Hoye T.R. (2017). Mechanistic Duality in Tertiary Amine Additions to Thermally Generated Hexadehydro-Diels–Alder Benzynes. Org. Lett..

[89] Arora S., Zhang J., Pogula V., Hoye T.R. (2019). Reactions of Thermally Generated Benzynes with Six-Membered *N*-Heteroaromatics: Pathway and Product Diversity. Chem. Sci..

[90] Arora S., Palani V., Hoye T.R. (2018). Reactions of Diaziridines with Benzynes Give *N*-Arylhydrazones. Org. Lett..

[91] Sneddon D.S., Hoye T.R. (2021). Arylhydrazine Trapping of Benzynes: Mechanistic Insights and a Route to Azoarenes. Org. Lett..

[92] Arora S., Sneddon D.S., Hoye T.R. (2020). Reactions of HDDA Benzynes with *C*,*N*-Diarylimines (ArCH = NAr’). Eur. J. Org. Chem..

[93] Zhang J., Hoye T.R. (2019). Divergent Reactivity during the Trapping of Benzynes by Glycidol Analogs: Ring Cleavage via Pinacol-Like Rearrangements vs. Oxirane Fragmentations. Org. Lett..

[94] Wang T., Oswood C.J., Hoye T.R. (2017). Trapping of Hexadehydro-Diels–Alder Benzynes with Exocyclic, Conjugated Enals as a Route to Fused Spirocyclic Benzopyran Motifs. Synlett.

[95] Ritts C.B., Hoye T.R. (2021). Sulfurane [S(IV)]-Mediated Fusion of Benzynes Leads to Helical Dibenzofurans. J. Am. Chem. Soc..

[96] Chen J., Palani V., Hoye T.R. (2016). Reactions of HDDA-Derived Benzynes with Sulfides: Mechanism, Modes, and Three-Component Reactions. J. Am. Chem. Soc..

[97] Palani V., Chen J., Hoye T.R. (2016). Reactions of Hexadehydro-Diels–Alder (HDDA)-Derived Benzynes with Thioamides: Synthesis of Dihydrobenzothiazino-Heterocyclics. Org. Lett..

[98] Watanabe T., Curran D.P., Taniguchi T. (2015). Hydroboration of Arynes Formed by Hexadehydro Diels–Alder Cyclizations with *N*-Heterocyclic Carbene Boranes. Org. Lett..

[99] Yun S.Y., Wang K.P., Lee N.-K., Mamidipalli P., Lee D. (2013). Alkane C–H Insertion by Aryne Intermediates with a Silver Catalyst. J. Am. Chem. Soc..

[100] Mamidipalli P., Yun S.Y., Wang K.-P., Zhou T., Xia Y., Lee D. (2014). Formal Hydrogenation of Arynes with Silyl C_β_–H Bonds as an Active Hydride Source. Chem. Sci..

[101] Lee N.-K., Yun S.Y., Mamidipalli P., Salzman R.M., Lee D., Zhou T., Xia Y. (2014). Hydroarylation of Arynes Catalyzed by Silver for Biaryl Synthesis. J. Am. Chem. Soc..

[102] Ghorai S., Lee D. (2017). Aryne Formation via the Hexadehydro Diels–Alder Reaction and their Ritter-Type Transformations Catalyzed by a Cationic Silver Complex. Tetrahedron.

[103] Ghorai S., Lin Y., Xia Y., Wink D.J., Lee D. (2020). Silver-Catalyzed Annulation of Arynes with Nitriles for Synthesis of Structurally Diverse Quinazolines. Org. Lett..

[104] Karmakar R., Ghorai S., Xia Y., Lee D. (2015). Synthesis of Phenolic Compounds by Trapping Arynes with a Hydroxy Surrogate. Molecules.

[105] Wang K.-P., Yun S.Y., Mamidipalli P., Lee D. (2013). Silver-Mediated Fluorination, Trifluoromethylation, and Trifluoromethylthiolation of Arynes. Chem. Sci..

[106] Karmakar R., Wang K.P., Yun S.Y., Mamidipalli P., Lee D. (2016). Hydrohalogenative Aromatization of Multiynes Promoted by Ruthenium Alkylidene Complexes. Org. Biomol. Chem..

[107] Xiao X., Wang T., Xu F., Hoye T.R. (2018). Cu^I^-Mediated Bromoalkynylation and Hydroalkynylation Reactions of Unsymmetrical Benzynes: Complementary Modes of Addition. Angew. Chem. Int. Ed..

